# Redox Homeostasis in Poultry: Regulatory Roles of NF-κB

**DOI:** 10.3390/antiox10020186

**Published:** 2021-01-28

**Authors:** Peter F. Surai, Ivan I. Kochish, Michael T. Kidd

**Affiliations:** 1 Department of Biochemistry, Vitagene and Health Research Centre, Bristol BS4 2RS, UK; 2 Department of Hygiene and Poultry Sciences, Moscow State Academy of Veterinary Medicine and Biotechnology named after K. I. Skryabin, 109472 Moscow, Russia; kochish.i@mail.ru; 3 Department of Biochemistry and Physiology, Saint-Petersburg State Academy of Veterinary Medicine, 196084 St. Petersburg, Russia; 4 Department of Microbiology and Biochemistry, Faculty of Veterinary Medicine, Trakia University, 6000 Stara Zagora, Bulgaria; 5 Department of Animal Nutrition, Faculty of Agricultural and Environmental Sciences, Szent Istvan University, H-2103 Gödöllo, Hungary; 6 Center of Excellence for Poultry Science, University of Arkansas, Fayetteville, AR 72701, USA; mkidd@uark.edu

**Keywords:** antioxidants, NF-κB, oxidative stress, poultry, redox balance

## Abstract

Redox biology is a very quickly developing area of modern biological sciences, and roles of redox homeostasis in health and disease have recently received tremendous attention. There are a range of redox pairs in the cells/tissues responsible for redox homeostasis maintenance/regulation. In general, all redox elements are interconnected and regulated by various means, including antioxidant and vitagene networks. The redox status is responsible for maintenance of cell signaling and cell stress adaptation. Physiological roles of redox homeostasis maintenance in avian species, including poultry, have received limited attention and are poorly characterized. However, for the last 5 years, this topic attracted much attention, and a range of publications covered some related aspects. In fact, transcription factor Nrf2 was shown to be a master regulator of antioxidant defenses via activation of various vitagenes and other protective molecules to maintain redox homeostasis in cells/tissues. It was shown that Nrf2 is closely related to another transcription factor, namely, NF-κB, responsible for control of inflammation; however, its roles in poultry have not yet been characterized. Therefore, the aim of this review is to describe a current view on NF-κB functioning in poultry with a specific emphasis to its nutritional modulation under various stress conditions. In particular, on the one hand, it has been shown that, in many stress conditions in poultry, NF-κB activation can lead to increased synthesis of proinflammatory cytokines leading to systemic inflammation. On the other hand, there are a range of nutrients/supplements that can downregulate NF-κB and decrease the negative consequences of stress-related disturbances in redox homeostasis. In general, vitagene–NF-κB interactions in relation to redox balance homeostasis, immunity, and gut health in poultry production await further research.

## 1. Introduction

Redox biology is a very quickly developing area of modern biological sciences, and roles of redox homeostasis in health and disease have recently received tremendous attention [1,2,3,4,5,6]. There are a range of redox pairs in cells/tissues responsible for redox homeostasis maintenance/regulation. They include, but are not limited to, NAD^+^/NADH, NADP^+^/NADPH, GSSH/GSH (glutathione system), Trx^ox^/Trx^red^ (thioredoxin system), protein thiols^ox^/protein thiols^red^. It is believed that redox signaling is tightly integrated with various homeostatic mechanisms [7] and all redox elements are interconnected and regulated by various means, including antioxidant and vitagene networks [1]. The redox status is responsible for maintenance of cell signaling and cell stress adaptation. There are a range of redox sensors which determine redox imbalance and activate various pathways for its re-establishment. Among them are proteins Keap1, an inhibitor of Nrf2, and IκB, an inhibitor of NF-κB, which have received a lot of recent attention. Indeed, oxidation of SH groups in Cys of Keap1 or phosphorylation of IκB are important triggers for nuclear translocation and activation of Nrf2 and NF-κB—important players in the redox homeostasis regulation [6]. In particular, a recent model suggests regulation of all collaborating metabolic organs in the body through changes in circulating redox metabolites [5].

The physiological roles of redox homeostasis maintenance in avian species, including poultry, are poorly characterized. However, for the last 5 years, this topic attracted a lot of attention, and a range of publications covered some related aspects. Indeed, the redox system imbalance is shown to be associated with protein oxidation and impaired quality of poultry meat [8,9]. In broilers, subjected to dietary and heat stress, magnesium supplementation was indicated to improve redox status and meat quality [10]. The influence of selenium and selenoproteins in maintaining redox balance and immune responses of poultry and pigs was presented [11], and the effect of oxidative stress and redox disbalance on inflammation, including a detailed immune system investigation, was discussed [12,13]. Oxidative stress-related disturbances of the redox balance in the poultry gut have also been described [13,14,15,16]. The long-term effects of Ochratoxin A on the glutathione redox system in chickens have been investigated [17], and the protective effects of milk thistle on redox-homeostasis imbalance of duck liver imposed by mycotoxins [18] were shown. Furthermore, the detrimental effects of heavy metals (e.g., As) on redox imbalance in chickens have been reported [19]. Nutritional modulation of the antioxidant capacities and redox homeostasis in poultry by selenium [13,20], vitamin E [21], and carotenoids [22], including astaxanthin [23], has been described. Recently, the vitagene concept of stress adaptation was developed, and questions related to redox balance maintenance in poultry under various stress conditions were addressed [1]. In fact, the vitagene family includes superoxide dismutase (SOD), heat shock protein 70 (HSP70), heme oxygenase 1(HO-1), elements of thioredoxin and glutathione systems, and sirtuins [24,25,26]. Indeed, induction/activation of the aforementioned genes leading to synthesis/expression of protective molecules helps animals/poultry adapt to stress by using their internal resources to the maximum extent.

Furthermore, transcription factor Nrf2 was shown to be a master regulator of antioxidant defenses via activation of various vitagenes and other protective molecules to maintain redox homeostasis in cells/tissues [1,27]. It was shown that Nrf2 is closely related to another transcription factor, namely, NF-κB, responsible for control of inflammation; however, its roles in poultry are not yet characterized. The importance of understanding the molecular mechanisms of redox homeostasis maintenance and the regulatory roles of NF-κB in this process is related to several important issues in poultry production. Firstly, intensive poultry production is related to a variety of stresses which cannot be avoided because of price-sensitive production of meat and eggs [28,29,30]. Secondly, commercial poultry production is based mainly on large production units where several hundred birds are kept in a single room. In such conditions, immune protection against various microbial and viral diseases becomes an important issue, and several vaccinations during the production period take place [31]. Therefore, the important roles of NF-κB in maintaining the high immunocompetence of commercial birds deserve more attention. Because of growth-promoting antibiotic prohibition in poultry production in many countries with developed poultry production, poultry farmers face a lot of challenges associated with gut health problems and immunosuppression [32,33], where NF-κB is known to be involved. The global poultry industry faces challenges from *Salmonella* contamination of meat and eggs [34,35,36] and *Campylobacter* contamination of chicken meat [37], and understanding NF-κB functioning in poultry may help to develop effective protective measures against the aforementioned pathogens. Avian flu is also a great challenge for the global poultry industry [38], and understanding the involvement of NF-κB in antiviral immunity is on the agenda of many research centers worldwide. Furthermore, the avian immune system has a range of differences from the mammalian immune system (e.g., absence of lymph nodes, different antibody repertoire, absence of myeloperoxidase in macrophages, etc.) [39,40,41] and, therefore, understanding the regulatory functions of NF-κB would help to design various approaches for immunomodulation [42]. Lastly, there are a range of inflammation-associated conditions in poultry (see Section 8) where the crucial role of NF-κB is well known [43,44].

Therefore, the aim of this review is to provide and describe a current view on the NF-κB system functioning in poultry as an important part of redox balance maintenance mechanisms with a specific emphasis on the nutritional modulation of NF-κB under various stress conditions. For this purpose, a literature search using key words “NF-κB” and “poultry” or “chicken” was conducted using PubMed and Web of Science. Results of the analysis of relevant papers were included into the review. Since we were not able to find any reviews related to the roles of NF-κB in poultry, published in peer-reviewed journals, we also provide general information about the structure and functions of NF-κB on the basis of recent publications in this area.

## 2. Transcription Factor Nuclear Factor Kappa B

Nuclear factor kappa B (NF-κB) was discovered in the lab of the Nobel Prize winner, David Baltimore, as an inducible transcription factor in lymphocytes [45]. In general, NF-κB structure, functions, and regulation have been extensively characterized in recent comprehensive reviews [46,47,48]; however, in this review, the major emphasis is given to oxidative stress and the roles of NF-κB in the regulation of adaptive homeostasis in avian species.

The NF-κB family consists of several transcription factors characterized by Rel-homology domains (RHDs) that are able to bind to specific DNA sequences known as κB sites located in the promoter and enhancer regions of various genes [49]. It has been found that NF-κB is conserved across different phyla, and, in mammalian and avian species, it consists of a group of five related proteins (subunits) that are capable of binding to DNA: p50 (a 50 kDa protein also known as NF-κB1), p52 (known as NF-κB2), p65 (also known as RelA), c-Rel, and RelB. In fact, p65, RelB, and c-Rel are characterized by a C-terminal transcription activation domain (TAD) that participates in the positive regulation of gene expression [50]. Interestingly, the other NF-κB family members, NF-κB1 and NF-κB2, were shown to be synthetized as larger precursor proteins (p105 and p100) with subsequent proteolytic processing to p50 and p52 [47]. Transcription factor subunits of NF-κB, for example, p65, can combine to form hetero- and homodimers of different composition, defined as the NF-κB complex. As a result of binding to a variety of DNA sequences called κB sites, NF-κB can effectively regulate different gene targets [51].

By using two independent LC–MS/MS experiments, 365 NF-κB/RelA-associated proteins were identified [52]. The functional categories enriched in the newly identified NF-κB/RelA-associated proteins identified by the authors include DNA-binding factors and enzymes, RNA-binding factors and enzymes, nuclear matrix and cytoskeleton components, ribosome biogenesis, protein degradation, and mitochondrial proteins. For the maintenance of redox balance and development of adaptive homeostasis, the last category related to mitochondrial proteins, including antioxidant proteins, namely, peroxiredoxins 3 and 1 (PRDX3, and PRDX1), is of great importance. Functional analysis of the newly identified RelA-binding proteins conducted by the authors confirmed the complexity of the NF-κB actions and its involvement in the regulation of important biological functions including regulation of protein ubiquitination, chromatin organization, response to DNA damage stimulus, post-transcriptional regulation of gene expression, cell-cycle progress, and microtubule cytoskeleton translation [52]. Interestingly, an update on NF-κB and proteomics published a year earlier [53] indicated that stimulation of specific receptors by RNA, DNA, or lipopolysaccharide (LPS) is associated with the interactions of a number of unique proteins regulating NF-κB expression and activity, which activate a range of genes creating an adaptive response. Indeed, their roles in the maintenance of redox balance and the creation of adaptive homeostasis via modulating transcription factors and vitagenes deserve more attention.

Dimerization takes place at a region named the RHD, which is essential for DNA binding, dimerization, and interaction with the inhibitory κB (IκB) proteins [54,55]. The p65/50 dimer is considered to be the most important dimer activating transcription. Hence, RelA-deficient mice are shown to be embryonically lethal as a result of liver apoptosis [56].

As a result of action by various stimuli, IκB proteins are known to be rapidly phosphorylated by IκB kinase (IKK) on specific serine residues (e.g., Ser-32 and Ser-36 of IκBα; Ser-19 and Ser-23 of IκBβ), followed by ubiquitination (by E2- and E3-ligases), and degradation by the 26S proteasome [57]. To make signaling more effective, on the one hand, NF-κB is shown to encode gene effectors potentiating and amplifying its activation in a feedforward fashion. On the other hand, NF-κB activation is associated with programming default feedback mechanisms responsible for its automatic termination (post mission completion) by regulating the amount/activity/expression of negative effectors including microRNAs (miRNAs), decoy receptors, and anti-inflammatory cytokines, which can lead to the inhibition of the signaling pathways, inhibitory proteins IκBα and IκBε, etc. [58,59,60].

There are some similarities in the regulation of Nrf2 and NF-κB in biological systems. For example, in physiological resting conditions, NF-κB is known to be located in the cytoplasm of cells in an inactive state tightly bound to the inhibitory IκB proteins (e.g., IκBα, IκBβ, IκBγ, IκBδ, IκBε, etc.) preventing its binding to target sites. It seems likely that the IκB proteins are responsible for masking the DNA-binding domain of NF-κB/REL proteins, leading to their sequestering in the cytoplasm. Interestingly, IκB proteins are responsible for checking and controlling the pathway due to nuclear export signals and their ability to remove NF-κB proteins from the nucleus [59].

**Figure 1:**
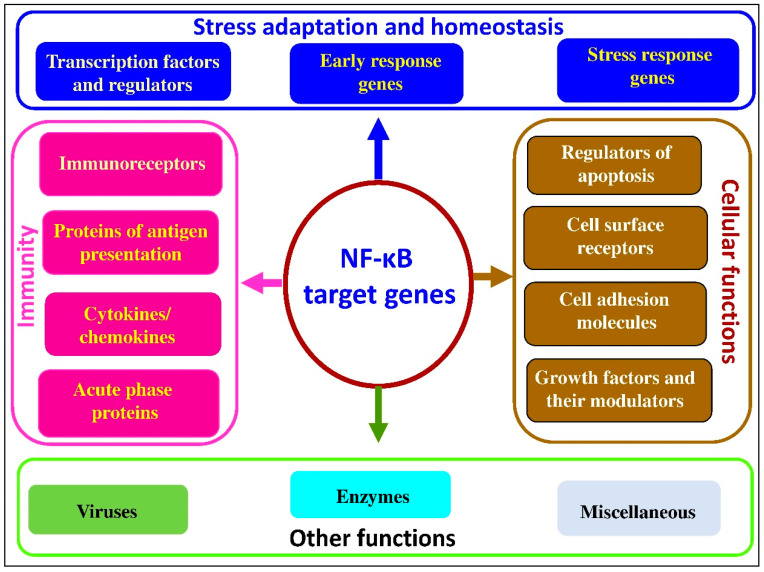
NF-κB target genes (adapted from [65]).

It seems likely that key steps in the NF-κB pathway include activation by the IκB kinase (IKK) complex, leading to the phosphorylation-induced proteasomal degradation of IκB proteins, dimer formation, and entering the nucleus, with subsequent binding to κB sites in promotor or enhancer regions of target genes [61]; along with other cofactors and histone acetyl transferases [62], it is also responsible for the transcription of more than 400 target genes regulating inflammation, immunity, apoptosis, stress adaptation, cell proliferation, and differentiation [63,64]. Major classes of target genes for NF-κB are shown in Figure 1.

As can be seen from the data presented in Figure 1, NF-κB target genes can be divided into several groups. The main group includes genes directly related to immunity, including immunoreceptors, proteins involved in antigen presentation, cytokines/chemokines and their modulators, and acute phase proteins. The second group of genes regulated by NF-κB are associated with stress adaptation and homeostasis maintenance under various stress conditions, including transcription factors and regulators, stress response genes, and early response genes. The third group of NF-κB-regulated genes are responsible for the regulation of various cellular function, including regulators of apoptosis, cell-surface receptors, cell adhesion molecules, growth factors, and their modulators. There are also NF-κB-regulated genes related to viruses, enzymes, and some other important signaling molecules. Therefore, the great variety of NF-κB-regulated genes explains the pivotal roles of this transcription factor in major physiological and pathophysiological processes in mammalian and avian species.

Generally, the NF-κB system is tightly regulated and can be activated by more than 15 pathways, with the two most common pathways being canonical (classical) and noncanonical (alternative) pathways [57]. The canonical pathway (i.e., the classical pathway) is based primarily on usage of p50 (the product of p105) in conjunction with p65 (p65/p50 nuclear dimer), IKKβ, and NF-κB Essential Modulator (NEMO), and it can be activated by pro-inflammatory cytokine receptors (interleukin 1 receptor, IL-1R and tumor necrosis factor receptor, TNFR), by pattern recognition receptors including the Toll-like receptors (TLRs), and by various genotoxic agents. It seems likely that the NF-κB stimulation by various external and internal stressors takes place via the canonical pathway. This includes a response to genotoxic and oxidative stresses, caused by ultraviolet radiation, ionizing radiation, reactive oxygen species (ROS), hypoxia, and dysfunctional mitochondria or endoplasmic reticulum by activating NF-κB IKK-dependently, IKK-independently, or both [60,66,67]. In particular, NF-κB can be activated by DNA damage via the canonical pathway [68].

The noncanonical pathway (known as the alternative pathway) is associated with p52 (the product of p100), RelB, NF-κB-inducing kinase (NIK), and IKKα and it is known to be triggered by a range of stimuli, including lymphotoxin B receptor, B-cell activating factor receptor 3, cluster of differentiation 40 (CD40), and receptor activator of NF-κB ligand (RANKL). Therefore, ligand-induced activation of the aforementioned receptors leads to the activation of NF-κB-inducing kinase (NIK), which specifically activates IKK1, inducing the phosphorylation and proteolytic processing of p100 to p52, followed by heterodimer formation with RelB to regulate target gene expression [56]. It is established that the noncanonical NF-κB pathway is deeply involved in regulation of the immune system, including creation of the adaptive immune response [69]. This includes regulation of B-cell development and function, including differentiation into long-lived antibody-producing plasma cells and memory B cells (for a review, see [46,70]), both being an integral part of the humoral immune response. Since, in comparison to mammals, avian spices are characterized by a different set of immunoglobulin (Ig) classes (IgD and IgE molecules are absent in birds) and a different cytokine repertoire [41], understanding the molecular mechanisms underlying regulation of the noncanonical NF-κB pathway in birds is a priority for avian scientists. Furthermore, a range of vaccinations used in poultry production are based on humoral response activation and memory B-cell formation [31,71], and the noncanonical NF-κB pathway could be a target for improvement of vaccination efficacy. It should be mentioned that the noncanonical NF-κB pathway was also shown to be involved in T-cell development in the thymus, and in orchestrating the formation and maintenance of effector and memory T cells [65]. Indeed, the cell-mediated immunity based on T-cell activity is of paramount importance for poultry, including resistance to viral diseases and vaccination efficacy [72,73].

It is important to mention that canonical and noncanonical NF-κB pathways interact with each other. For example, in the classical NF-κB pathway, the first protein transcribed is IκBα. Therefore, it is believed that, in order to inhibit further transcription and restore the original latent state of NF-κB signaling, newly synthesized IκBα can enter the nucleus, remove NF-κB from DNA, and export the complex back to the cytoplasm [49]. Interestingly, the canonical NF-κB pathway is considered to be antiapoptotic, while the noncanonical pathway is proapoptotic [50].

It well known that NF-κB responds to a large variety of external and internal stress signals/stimuli including oxidative stress [62], playing essential roles in the development and maintenance of tissue homeostasis by regulating the transcription of an array of different genes, including proinflammatory cytokines, as well as adhesion molecules, antimicrobial peptides, and acute phase proteins [74,75,76]. In fact, NF-κB signaling can be considered as an emergency response system, since activation of NF-κB was shown to occur very quickly (within minutes) as a result of release from IκB or as a consequence of cleavage of the inhibitory ankyrin repeat domains of p100 and p105 [50]. Under physiological conditions, the majority of NF-κB-activated genes regulate biological processes associated with cell growth, protection, and repair. They are involved in T-cell maturation, DNA damage repair, tissue healing after injury, and orchestrating the fight against infections [60,67]. Indeed, it has been proven that activation of NF-κB is an evolutionarily conserved, effective mechanism of host defense against infection and stress [77]. However, excessive NF-κB activation in commercially relevant stress conditions in poultry and farm animal production systems can lead to detrimental consequences, including chronic inflammation, compromised health status, and decreased productive and reproductive performance. The repertoire of stimuli implicated in the NF-κB activation is very diverse and also includes inhibitory κB kinases, cell-surface receptors, and NF-κB-inducible inhibitor proteins (IB proteins), as well as factors regulating the post-translational modification of the Rel proteins, etc. [63,64,74,75,76]. Furthermore, p65 and p50 are the targets of many other post-translational modifications such as ubiquitination, acetylation, methylation, phosphorylation, oxidation/reduction, and prolyl-isomerization, leading to a change in NF-κB transcriptional activity due to affecting the interaction with DNA or as a result of changes in the protein–protein association of NF-κB [78].

**Figure 2:**
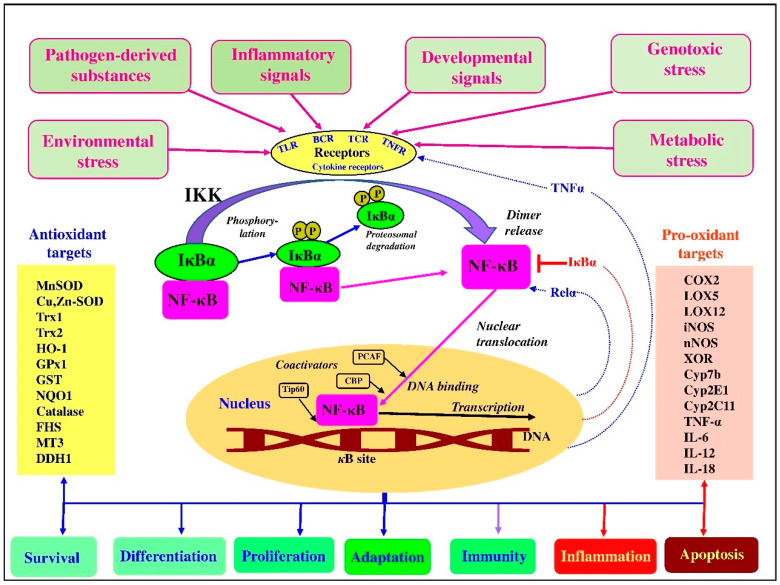
Activation of NF-κB and regulation of downstream transcriptional antioxidant and pro-oxidant targets in the canonical pathway (adapted from [1,59,80,81,82,83,84]).

NF-κB signaling in numerous cell types is involved in the development of various metabolic disorders. In particular, it is thought that resident tissue cells activate NF-κB in response to stress associated with nutrient excesses [58]. Furthermore, oxidized lipids in the bloodstream can induce NF-κB in vascular endothelia, while, in adipocytes, hepatocytes, and neurons, NF-κB is induced by metabolic or oxidative stress in the ER due to overnutrition. Furthermore, an excess of free fatty acids could also activate NF-κB via TLR4 [58]. Some examples of activation of NF-κB associated with regulation of downstream transcriptional antioxidant and pro-oxidant targets in the canonical pathway are shown in Figure 2.

Depending on physiological context, the activation of NF-κB can have different consequences. Indeed, NF-κB does not function alone but is part of various networks, including crosstalk with other transcription factors (Nrf2; signal transducer and activator of transcription 3, STAT3; Forkhead box O3, FOXO3; etc.), upstream kinases, sirtuins, Wingless-related MMTV integration site 4 (Wnt4), ROS, p53, and miRNAS, which determine the pattern of its effects on the expression of a battery of various genes [48,60,67]. Furthermore, there are regulatory mechanisms coordinating NF-κB association with various important pathways [50]. It has been suggested to consider NF-κB as a stress response factor, since NF-κB signaling is condition-dependent, and NF-κB-dependent cell death or survival would depend on the stimulus and the cell type involved. It seems likely that this complexity is responsible for many apparent contradictions in the literature [79]. However, most research data indicate that NF-κB signaling pathway enables cells to maintain homeostasis and survive under various stress conditions, including genotoxic stress [60,67].

NF-κB is involved in the modulation of many different molecular events, including inflammation, immune function, cellular growth, and apoptosis [46,70]. There are a range of NF-κB activators, including pathogen-derived substances (LPS) and inflammatory signals (TNF-α, IL-1), as well as other signals recognized by various receptors, including TNFRs, TLRs, T-cell receptors (TCRs), B-cell receptors (BCRs), and cytokine receptors, which lead to an activation of IκB kinase (IKK) with subsequent phosphorylation of NF-κB inhibitor. This leads to proteasomal degradation of IκB. As a result, the released NF-κB migrates into the nucleus and binds with its corresponding DNA-responsive elements in the presence of coactivators. This results in the transcription of antioxidant (anti-inflammatory) or pro-oxidant (proinflammatory) mediators [46,85]. It is believed that p65 can induce the expression of both negative regulators (IκBα, IκBε, etc.) and positive regulators (Relα, TNFα, etc.) participating in tuning the NF-κB pathway [83]. It is important to mention that NF-κB can be directly activated or inhibited by ROS in a context-dependent manner, including levels of ROS, exposure, and cell type [59,86,87]. Indeed, ROS-mediated oxidation of redox-sensitive cysteine residues of NF-κB subunits was shown to have dual effects (inducing or inhibiting) on the NF-κB signaling depending on the level of ROS, the cell type, and the types of stimuli [81,88]. On the one hand, ROS can activate the NF-κB pathway by imposing disulfide bond formation between Cys54 and Cys347 in ΙΚΚγ [89]. On the other hand, ROS can have an opposite effect: inhibiting NF-κB activation as a result of restricting IκBα degradation, due to inactivation of the proteasome [90].

The NF-κB system integrates diverse upstream input signals (from various stresses to pathogen-related molecules) recognized by various receptors into varied downstream output responses. This function is mediated via promotion of the expression of a variety of genes responsible for the synthesis of antioxidant or prooxidant molecules, improving antioxidant defenses and redox homeostasis. Alternatively, NF-κB activation can also lead to synthesis of proinflammatory cytokines, imposing inflammation and causing detrimental health- and production-related consequences in poultry and farm animals. In particular, NF-κB and STAT3 regulate common processes and share regulatory binding sites of antiapoptotic, cell cycle and proliferation, tissue resistance, and repair genes. Furthermore, hypoxia-inducible factor (HIF) and NF-κB share common activating stimuli, regulators, and targets [61].

## 3. NF-κB and Oxidative Stress

Free-radical production is considered to be an important process in biological systems responsible for the antibacterial action of oxidative burst in phagocytes, cell signaling, and stress adaptation [7]. However, an excess of reactive oxygen and nitrogen species (RONS) due to high level of stress or a compromised antioxidant system leads to damages to major biological molecules (proteins, polyunsaturated fatty acids (PUFAs), DNA, etc.) associated with immunosuppression, gut health problems, and decreased productive and reproductive performance of poultry [30]. Therefore, a variety of protective mechanisms have been developed during evolution to deal with RONS excess, and many transcription factors are involved in this process via regulating vitagenes and a myriad of antioxidant enzymes in stress conditions [1].

**Figure 3:**
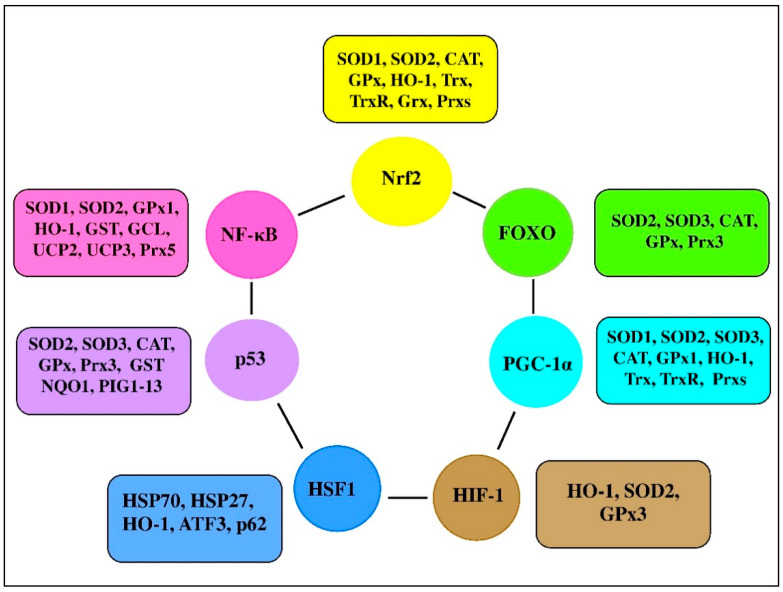
Transcription factors and their clients involved in redox homeostasis regulation (adapted from [1,92,93,94]).

There are a range of transcription factors acting cooperatively with NF-κB. For-example, NF-κB and STAT3 are shown to regulate common pathways and share regulatory binding sites of various protective genes, while HIF and NF-κB are reported to share common activating stimuli, regulators, and targets [61]. Indeed, the redox balance is believed to be orchestrated by a range of transcription factors, including Nrf2, NF-κB, activator protein 1 (AP-1), FoxO, peroxisome proliferator-activated receptors (PPARs), peroxisome proliferator-activated receptor-gamma coactivator 1α (PGC-1α), p53, and mitogen-activated protein kinase (MAPK; Figure 3 [91,92]). It seems likely that transcription factors and vitagenes are involved in the regulation of redox status by effectively modulating the expression and activity of ROS-generating enzymes and antioxidant enzymes [93].

NF-κB has long been considered to be a prototypical proinflammatory signaling pathway stimulating the immune system in response to various stimuli, including physical, physiological, and/or oxidative stress. For example, NF-κB is a key target in receptor-independent hypothalamic microinflammation [95] associated with intracellular organelle stress, including RNA stress response [96], endoplasmic reticulum (ER) stress [97], and defective autophagy [98]. NF-κB is involved in the regulation of many important physiological processes; however, its overactivation has been shown to be associated with increased risk of disease, while NF-κB suppression is associated with risk reduction [63]. Taking the former into account, understanding the role of NF-κB signaling in stress adaptation awaits further investigation. For example, HO-1 can improve cell protection from apoptosis by stimulating free heme catabolism. Interestingly, the HO-1 promoter region contains an NF-κB responsive element and, therefore, HO-1 expression is regulated by NF-κB, as well as by other transcription factors [99]. A central role for NF-κB in regulating mitochondrial respiration has been suggested [100]. In fact, by controlling the balance between glycolysis and respiration for energy provision, NF-κB is involved in energy homeostasis and metabolic adaptation [101]. The authors suggested to consider NF-κB as an important checkpoint connecting cell activation and proliferation with energy sensing and metabolic homeostasis. Since mitochondria are the main ROS source in the cell, it could be that NF-κB signaling is involved in the regulation of ROS formation, detoxification, and the maintenance of redox homeostasis.

## 4. Nrf2 and NF-ĸB Interplay in Oxidative Stress

Proof of the interaction and cooperative action of Nrf2 and NF-κB was taken from experimental work with various model systems employing plant extracts, individual compounds in vitro and in vivo, pure chemicals, and some known toxicants [6]. In our recent review, a central role of Nrf2 in antioxidant defenses and vitagene regulation was described in detail [27], and it seems likely that, under oxidative stress, the transcription factors NF-κB and Nrf2 antagonize each other to coordinate a stress response [60,67,76]. For example, deletion of Nrf2 (Nrf2 knockout mice) enhanced inflammation, while Nrf2 upregulation was reported to decrease NF-ĸB-dependent proinflammatory and immune responses [62]. In fact, several known Nrf2 activators are able to inhibit the NF-κB pathway. There are many examples showing that activation and repression occur between members of the Nrf2 and NF-κB pathways through various mechanisms [102]. Some mechanisms of Nrf2–NF-κB interactions are summarized in Table 1.

There is accumulating evidence indicating that various nutrients with antioxidant (AO) activities could differently affect transcription factors: increasing expression of Nrf2 and simultaneously decreasing NF-κB expression and activity. This was proven in various model systems employing plant extracts and individual polyphenolics. Firstly, in in vitro systems, these include sinomenine [120], aloin [121], cannabisin F [122], urolithin B [123], 4-ethylguaiacol [124], gambogic acid [125], and the combination of ascorbic acid and rutin [126]. There are also a range of in vivo studies confirming that various plant-derived compounds, mainly polyphenols, decrease NF-κB and increase Nrf2 activity/expression in various model systems. These include peiminine [127], hesperetin [128], oxyresveratrol, resveratrol and mulberroside [129], salvianolic acid A [130], naringenin [131], δ-amyrone [132], chrysin, luteolin, apigenin, hesperetin and 3′, 4′-dimethoxy hesperetin [133], luteoloside [134], alpinetin [135], amygdalin [136], rosmarinic acid [137], chiisanoside [138], arbutin [139], and chicoric acid [140]. Moreover, the different direction of activation of Nrf2 and NF-κB was also shown to be a result of exposure to various toxic compounds. However, other agents and stimuli, including, but not limited to, ROS, LPS, flow shear stress, oxidized low-density lipoprotein, and cigarette smoke, were reported to activate both Nrf2 and NF-κB pathways [101,102].

The redox outcome of the NF-κB–Nrf2 interaction would depend on the activation/inhibition of various antioxidant and prooxidant enzymes. It is known that some antioxidant enzymes are dependent on both Nrf2 and NF-κB. For example, expression of HO-1 is shown to be regulated by Nrf2, NF-κB, and HIF-1α signaling [60,67]. On the one hand, HO-1 was shown to possess a functional ARE that is activated by Nrf2 [141]. On the other hand, HO-1 was shown to have a functional NF-κB site [142]. HO-1 is known to be the stress-inducible enzyme providing AO protection in vertebrate systems, participating in the maintenance of redox balance and being responsible for adaptation to oxidative, inflammatory, and cytotoxic stress [25]. Similarly, a key catalytic subunit of glutamate-cysteine ligase, the key enzyme of the cellular GSH biosynthetic pathway, also has an ARE and can be activated by Nrf2 [143], whereas it also possesses a κB site and can be induced by NF-κB [144]. Since GSH is a key physiological buffer responsible for the redox homeostasis [145], regulation of its synthesis via Nrf2 and NF-κB pathways is of great importance for redox homeostasis maintenance related to high immunocompetence. Furthermore, MnSOD is also a target for both NF-κB [146] and Nrf2 [27]. It is well established that MnSOD, a key enzyme of the first line of the AO network, is located in mitochondria and deals with major biological ROS, namely, superoxide radicals, and it is considered to be a major player in the establishment and maintenance of redox homeostasis [30]. Furthermore, glutathione peroxidase 1 (GPx1) and glutathione S-transferase (GST) expression and activities are also under strict control by NF-κB [81] and Nrf2 [13]. The important roles of these AO enzymes in AO defense and redox homeostasis have been previously discussed [13]. It seems likely that another redox balance regulator, namely, thioredoxin, is also regulated by NF-κB [146,147] and Nrf2 [25]. It is interesting that HO-1, SOD, and thioredoxins belong to the vitagene family responsible for stress adaptation and redox homeostasis [24].

It should also be mentioned that the NF-κB pathway can induce free-radical production via activating ROS-producing enzymes, including NADPH oxidase [148], cyclooxygenase-2 (COX-2) [102], cytochrome p450 enzymes, inducible nitric oxide synthase (iNOS), neuronal NOS (nNOS), and xanthine oxidase/dehydrogenase [81]. The impact of such ROS production on redox balance and adaptation to stress is still not well established; however, this complicates the interpretation of results related to NF-κB–Nrf2 interactions in biological systems under various stress conditions.

In many cases, activation of various transcription factors, including Nrf2, NF-κB, AP-1, HIF-1α, p53, PPAR-γ, and *β*-catenin/Wnt, was associated with the oxidative stress [149]. Therefore, a complex crosstalk between Nrf2 and NF-κB pathways under various stress conditions [62] further complicates interpretation of results related to the relative impact of each pathway on the regulation of stress adaptation. Indeed, as mentioned above, Nrf2 and NF-κB affect each other’s expression and activity to coordinate antioxidative and inflammatory responses; however, molecular mechanisms of this interconnection are not yet known [62].

**Figure 4:**
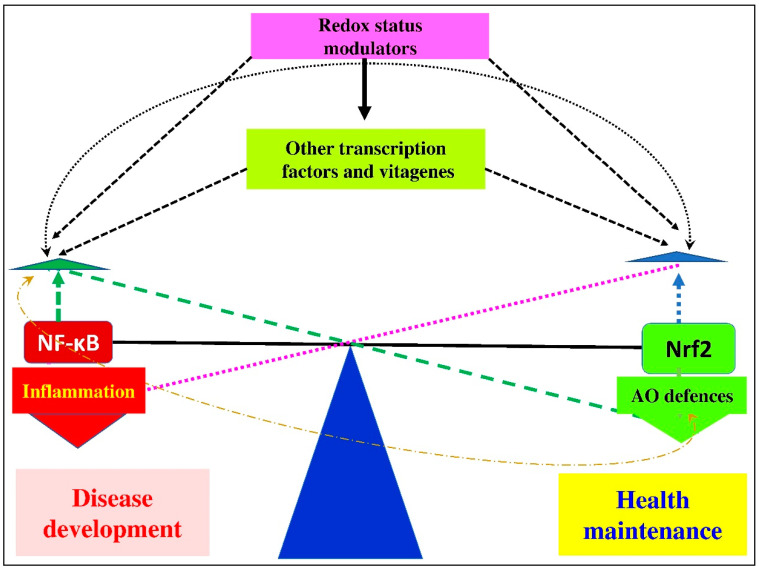
Hypothetical Nrf2–NF-κB crosstalk [1,27].

It is believed that condition-dependent, stress-associated changes in redox balance and in expressions/activities of transcription factors (e.g., Nrf2/Keap1 and NF-κB/IκB/IKK) are responsible for providing adaptive cell responses to a variety of stress stimuli through orchestrating the optimal expression of protective target genes [150]. A hypothetical scheme of the Nrf2–NF-κB crosstalk is shown in Figure 4.

In physiological conditions, a delicate balance between Nrf2 and NF-κB expression in various tissues is well coordinated and maintained. It seems likely that increased NF-κB expression as a result of low/moderate stresses can lead to a simultaneous increase in the expression of Nrf2, leading to improved antioxidant defenses. At the same time, decreased NF-κB expression can be observed as a feedback mechanism. This balance is also regulated by other transcription factors and vitagenes. In the case of high oxidative stress, when the ability of the AO defense network to deal with RONS production is overwhelmed, the Nrf2/NF-κB balance would be broken. In such conditions, redox status would be compromised with detrimental consequences to animal health. Furthermore, the productive and reproductive performance of poultry and farm animals would be decreased.

## 5. NF-κB in Poultry Production

The regulatory roles of NF-κB in poultry are still poorly understood, but accumulating information clearly indicates that, similar to mammals, NF-κB is a main regulator of many important processes, including inflammation in avian species. In 1993, complementary DNA (cDNA) clones encoding the chicken NF-κB p65 subunit were isolated, and, according to the information provided by the authors, chicken NF-κB can be briefly characterized as follows [151]:

- Chicken p65 was shown to be approximately 55% identical to the mouse and human p65 proteins. Similar to its mammalian counterpart, chicken p65 contains the Rel homology domain (RHD) in its N-terminal consisting of 286 amino acids and the putative transactivation domain in its C-terminal region;
- It was proven that the RHD was highly conserved between the chicken and mammalian p65 proteins;
- The highest expression of a 2.6 kb transcript of p65 was detected in the spleen. It was also detected in other organs;
- A fusion protein containing the RHD of chicken p65 was reported to bind to a consensus kappa B-site;
- p65 was shown to form one or more complexes with various cellular proteins, including p50, p105, and c-Rel in chicken spleen cells [151].

Furthermore, the cDNA clones encoding chicken p50B/p97 were isolated [152]. The amino-acid sequence of the precursor protein p97 was found to be characterized by a conserved structure. In particular, it was shown to have 86% identity in the RHD and lower (56%) identity in the ankyrin repeat domain (ARD) to human p50B/p97. Similar to previous findings, expression of this gene was also found to be highest in the chicken spleen [152]. In 1995 from a chicken genomic library, a clone containing the avian I kappa B-alpha gene was isolated [153]. Main characteristics of I kappa B-alpha can be summarized as follows: recognizable promoter elements (i.e., TATA and CAAT boxes) were not found in avian I kappa B-α. There were seven putative Rel/NF-kappa B binding sites in avian I kappa B-α. When transfected into cells which produce I kappa B-α, a CAT reporter construct containing the 5′ upstream region of I kappa B-α was expressed. The regulatory elements promoting I kappa B-α expression were identified within 1000 nt of the transcription start site. I kappa B-alpha was shown to be found as a single-copy gene per haploid genome. This gene was expressed in avian hematopoietic tissues and in lymphoid cells transformed by avian reticuloendotheliosis virus [153]. It was suggested that, similar to mammals, in chicken, p65 and c-Rel comprise components of the protein complexes that are able to bind to the kappa B-like sequence. This binding could lead to the progressively activated expression of the chicken lysozyme gene observed during the terminal differentiation of macrophages [154].

In 2001, Piffat et al. constructed and characterized a composite cDNA encoding most of the chicken RelB transcription factors [155], and their results can be summarized as follows: within the RH domain, chicken RelB (cRelB) protein was characterized by a high degree of sequence similarity to other vertebrate RelB proteins. However, outside this domain, cRelB was substantially less conserved. cRelB was found to be more widely expressed than mammalian RelB, and it was identified to have functional properties similar to other vertebrate RelB proteins. cRelB was reported to be unable to bind DNA in a homodimer form; however, it could form DNA-binding heterodimers with NF-kappaB p50 or p52. Overexpressed cRelB was shown to be present in the nucleus in chicken embryo fibroblasts. The nonconserved C-terminal sequences of cRelB contained a transactivation domain found in chicken and mouse fibroblasts [155]. A new isoform of chicken myeloid differentiation factor 88 (MyD88-2) expression was detected in a range of tissues tested and its overexpression was found to significantly induce the activation of NF-κB in vitro [156]. Recently the duck IKKα (duIKKα) gene was cloned and characterized. In fact, DuIKKα was reported to encode a protein containing 757 amino acids and having high sequence identities with the goose IKKα. Duck liver and heart were characterized by a high expression of duIKKα messenger RNA (mRNA), while its expression was reported in all tested tissues, including muscular stomach, spleen, heart, liver, lung, kidney, cerebellum, cerebrum, windpipe, muscle, glandular stomach, thymus, duodenum, cecum, pancreas, and bursa of Fabricius [157]. An important role of du IKKα in NF-κB regulation has been demonstrated by increasing or inhibiting expression of duIKKα. On the one hand, overexpression of duIKKα was shown to substantially increase NF-κB activity with subsequent induction of cytokines interferon beta (IFN-β), IL-1β, IL-6, and IL-8 in duck embryo fibroblasts. On the other hand, knockdown of duIKKα was found to significantly decrease LPS-, poly(I:C)-, poly(dA:dT)-, duck enteritis virus (DEV)-, or duck Tembusu virus (DTMUV)-induced NF-κB activation [157]. It seems likely that IKKα is evolutionarily conserved. In fact, phosphorylation of Ser176 and Ser180 in the active center of IKKα is believed to be vital to IKKα activation, and those Ser residues were shown to be well conserved among mammals, birds, and fish [157].

It was shown that the NF-κB family of transcription factors contribute to activation-induced cytidine deaminase-mediated gene conversion in chickens [158]. Gallus heat-shock cognate protein 70 was shown to regulate RelA/p65 gene expression induced by Apoptin, a nonstructural protein of chicken anemia virus [159]. In chicken heterophils, bacterial TLR agonists were indicated to activate NF-κB-mediated leukotriene B4 and prostaglandin E2 production [160]. A switchlike response in NF-κB activity is based on the existence of a threshold in the NF-κB signaling module, and phosphorylation of the Ser-578 residue of the scaffolding protein caspase recruitment domain (CARD)-containing protein 1 (CARMA1) was shown to account for the feedback [161]. It is known that tumor necrosis factor receptor-associated factors (TRAFs) are responsible for activation of various signaling cascades, being key regulatory proteins in NF-κB signaling pathways [162]. It seems likely that avian TRAFs play important roles in defending against both RNA and DNA virus infection. In fact, chicken TRAF3 (chTRAF3) was shown to encode a protein of 567 amino acids with high identity to TRAF3 homologs from mammals being abundantly expressed in the spleen, thymus, lung, and small intestine [163]. Of note, the authors showed that Newcastle disease virus F48E9 challenge was responsible for TRAF3 suppression in chicken embryo fibroblast cells. Recently, the full-length duck TRAF6 (duTRAF6) cDNA from embryo fibroblasts was cloned, and it was shown that duTRAF6 was widely expressed in different tissues. Interestingly, overexpression of duTRAF6 was found to activate NF-κB and induce interferon-β expression [164]. It has been shown that goose TRAF6 shared similar features with the TRAF6 of other avian species, being an essential regulator for inducing the activity of NF-κB and playing important roles in innate immune response [165]. The amino-acid sequence of pigeon FRAF6 (piTRAF6) was shown to share a strong identity with that of other birds. Furthermore, piTRAF6 expression was shown in all examined tissues, including heart, lung, spleen, thigh muscle, large intestine, caecum, kidney small intestine, brain, bursa of Fabricius, rib, and muscular stomach [166]. The heart was characterized by the highest level of piTRAF6 transcript, and the muscular stomach had the lowest level of piTAF6 transcript. On the one hand, overexpression of piTRAF6 was shown to induce NF-κB in a dose-dependent manner with increased IFN-β expression. On the other hand, piTRAF6 knockdown was reported to suppress NF-κB activation in HEK293T cells [166]. Furthermore, the pigeon TRAF3 (PiTRAF3) gene was reported to be highly expressed in the spleen, lung, kidney, brain, thymus, and muscle, while a moderate expression was observed in the small and large intestines, with relatively weak expression in the heart and liver [167].

Among the five major families of pattern recognition receptors (PRRs), Toll-like receptors (TLRs) and nucleotide-binding oligomerization domain (NOD)-like receptors (NLRs), in particular, NOD1, recently received major attention in relation to their roles in avian immunity via NF-κB regulation. Indeed, NF-κB is considered to be the major transcription factor involved downstream of the TLR signaling pathway [168]. Avian TLRs are shown to be different from their mammalian counterparts: absence of TLR8 and TLR9, along with presence of TLR1La, TLR1Lb, TLR15, and TLR21 [169]. Therefore, in chickens, 10 TLR receptor genes were identified: TLR1LA, TLR1LB, TLR2B, TLR2A, TLR3, TLR4, TLR5, TLR7, TLR15 [170], and TLR21 [171]. Among them, TLR1LA, TLR1LB, TLR2A, TLR2B, TLR4, TLR5, and TLR15 are responsible for bacterial component (lipoproteins, peptidoglycans, LPS, and flagellin) detection, while TLR3 and TLR7 detect viruses (double-stranded RNA (dsRNA), single-stranded RNA (ssRNA), imidazoquinoline compounds), and TRL21 detects CpG oligodeoxynucleotides in viruses and bacteria [171]. Initially, it was reported that chicken TLR2 and TLR4 can mediate LPS-stimulated oxidative burst, while CD14 and TLR2 are involved in the mediation of lipoteichoic acid-stimulated oxidative burst in heterophils [172]. The tissue-specific expression of chicken TLRs (TLR2A, TLR3, TLR4, TLR5, TLR7, TLR15, and TLR21) during embryonic development was evaluated and early (third embryonic day) expression of all the TLR mRNAs was reported [173]. Furthermore, TLR1 (type 1 and 2), TLR2 (type 1 and 2), and TLRs 3–5, 7, 15, and 21 were shown to be expressed in the chicken follicular theca. The connection of the TLRs to NF-κB was proven experimentally; the expression of IL-1β, IL-6, chemotactic and angiogenic factor (CXCLi2), and IFN-β in tissues incubated with LPS was downregulated by an inhibitor of NF-κB [168].

It seems likely that NF-κB is involved in the activation of avian antimicrobial peptides. For example, chicken intestine defensins (e.g., AvBD13) were suggested to be endogenous ligands for TLR4 able to enhance the proliferation of monocytes via the NF-κB pathway [174]. It should be mentioned that cathelicidins (CATHs), short cationic host defense peptides, also act in close concert with NF-κB. Indeed, in macrophages primed by LPS, pigeon CATH2 was shown to act through MAPK and NF-κB signaling pathways to enhance expression of the anti-inflammatory cytokine, while downregulating the expressions of inducible nitric oxide synthase and proinflammatory cytokines and inhibiting the TLR4 pathway [175]. Furthermore, NK-lysin/granulysin (NKL), an antimicrobial cationic peptide expressed in natural killer cells and cytotoxic T lymphocytes, was identified in different avian species, including chicken, turkey, zebra finch, and quail, and the 5′ flanking region of quail NKL was shown to contain two NF-κB-binding sites [176], suggesting participation of NF-κB in regulation of NKL activity.

In hen vaginal cells, NF-κB was shown to be the transcription factor responsible for the expression of various proinflammatory cytokines and chemokines. In fact, in response to the ligands of TLR3, 4, and 21, increased expression of IL1B, IL6, and CXCLi2 was observed, while IL1B expression was found in response to the ligands of TLR5 and 7 [177]. The authors suggested that NF-κB-dependent expression of cytokines might provide the important defense capability of vaginal tissue to bacterial and viral infections. Activation of TLR3 was shown to induce the expression of NF-κB and the production of type-I interferon [178]. IFN-κ (a type I IFN) in both chicken and duck was found to be constitutively expressed in a range of tissues, including spleen, skin, lung, and peripheral blood mononuclear cells (PBMCs), and it could be induced after treatment with virus in PBMCs [179]. The duck TLR4 (duTLR4) gene was shown to be strongly expressed in the liver, kidney, spleen, intestine, and brain [180].

Goose TLR3 was shown to be analogous to mammalian TLR3 and recognized double-stranded RNA with subsequent activation of NF-κB [178]. In fact, the goose TLR3 gene was shown to encode a protein containing 896 amino acids, sharing 46.7–84.4% homology with other species with highest expression in the pancreas and lowest in the skin. The authors showed that geese infected with H5N1 were characterized by significant upregulation of TLR3 in various tissues, including the lung and brain [178]. The goose TLR5 (gTLR5) gene was shown to be expressed in all studied tissues, including high expression in the liver, spleen, and brain, moderate expression in kidney, lung, heart, bone marrow, small intestine, large intestine, and PBMCs, and minimal expression in the cecum [181]. It was also shown that gTLR5 can detect flagellin from *Salmonella* Typhimurium with subsequent NF-κB activation in HEK293 cells. It seems likely that there is a tissue-specific regulation of TLR expression in the process of orchestrating the immune response against bacterial pathogens [181]. Goose TLR2-1 was also shown to play an important role in the recognition of *Mycoplasma fermentans* lipopeptide, Mycoplasma gallisepticum (MG) and Salmonella enteritidis (SE), and it induced the activation of NF-κB [182]. Furthermore, in HEK293T cells, flagellin was shown to induce pigeon NF-κB via TLR5 activation. This was associated with significant upregulation of IL-1β, IL-8, TNF-α, and IFN-γ. Importantly, the levels of TLR5, NF-κB, IL-6, IL-8, chemokine ligand 5 (CCL5), and IFN-γ mRNA were significantly upregulated as a result of flagellin stimulation of pigeon splenic lymphocytes. As could be expected, goose TLR5 knockdown was shown to be associated with the significantly downregulated expression of NF-κB and related cytokines/chemokines [183]. Interestingly, the antiviral activity of pigeon IFN-α is believed to depend on the expression of NF-κB [184]. It is known that single-stranded viral RNAs and antiviral imidazoquinoline compounds can be recognized by TLR7 with subsequent NF-κB activation. Recently, it was shown that, in pigeon, agonist R848 (imidazoquinoline) can activate NF-κB via TLR7 [185].

It seems likely that chicken NOD1 activation in response to pathogenic invasion is of great importance for immune defense. In partridge chicken, NOD1 was shown to be widely distributed in various tissues, with the highest expression found in testes. Of note, as a result of *S. enterica* serovar Enteritidis infection, induced expression of chNOD1, as well as the effector molecule NF-κB, was observed in the spleen tissue [186]. Duck NOD1 (duNOD1) was shown to be widely distributed in various organs, including heart, liver, spleen, lung, kidney, cerebrum, cerebellum, colon, glandular stomach, thymus, and bursa of Fabricius tissue with the highest expression found in the liver. Of note, duNOD1 overexpression induced NF-κB, TNF-α, and IL-6 activation in duck embryo fibroblasts (DEFs), while silencing duNOD1 was indicated to decrease the activity of NF-κB in stimulated DEFs [187].

Chicken IL-26 was shown to regulate immune responses through the NF-κB and the Janus kinase (JAK)-signal transducer and activator of transcription (STAT) Janus kinase signaling pathways [188]. Similarly, chicken IL-11 was shown to bind to IL-11R and activated the NF-κB, JAK/STAT, and MAPK signaling pathways, leading to modulation of T helper 1 (Th1)/Th17 and Th2 cytokine production in chicken cell lines [189]. Chicken interleukin-17B was shown to induce the NF-κB signaling pathway, leading to increased expression of proinflammatory cytokines playing a critical role in host defense against the bacterial pathogens [190]. In eukaryotic and prokaryotic expression systems, recombinant chicken TNF-α was generated to demonstrate its biological activity. In particular, as a result of binding to TNF-α receptor 1, the cytokine was shown to induce a complex signaling cascade leading to induction of the classical NF-κB pathway [191].

In Gaoyou duck skeletal muscle (*Anas platyrhynchos domesticus*), NF-κB motifs (binding sites) were identified, which are believed to be responsible for transcriptional regulation of the slow skeletal muscle troponin I (TNNI1) gene [192]. It seems likely that chicken NF-κB plays a central role in antiviral defense. In fact, chicken tracheal epithelial cells were shown to initiate effective antiviral responses after stimulation with TLR ligands as a result of interferon regulatory factor 7 (IRF7) and NF-κB signaling pathways associated with activation of other cells, such as macrophages [193].

Receptor activator of NF-κB ligand (RANKL), a new member of the chicken TNF superfamily, was recently identified and characterized [170]. Therefore, chicken RANKL (chRANKL), sharing ~59–62% identity with mammalian RANKL, was shown to be ubiquitously expressed in chicken tissues. In nonlymphoid tissues, chRANKL mRNA expression levels were shown to be highest in muscle, while, in lymphoid tissues, the highest RANKL expression was found to be in the thymus, followed by the upper gut and the bone marrow [194]. Recently identified and functionally characterized chicken leukocyte immunoglobulin-like receptor A5 (LILRA5) was reported to activate/induce NF-κB, as well as other immunoregulatory pathways [195].

## 6. Effect of Various Stress Factors on NF-κB Expression and Activity in Poultry

### 6.1. Thermal Stress

Continuous exposure of farm animals to an acute or gradual rise in habitat temperature was shown to induce oxidative stress leading to reduced survivability and longevity [196], reduced growth, decreased productive and reproductive performance, and compromised health in poultry [197,198]. Intestinal damages due to thermal stress could lead to redox balance disturbances and inflammatory reactions regulated via NF-κB [12]. It seems likely that NF-κB expression in thermally stressed birds is condition-dependent, including temperature, exposure duration, and bird’s age. On the one hand, when quails at the age of 20 weeks were heat-stressed (34 °C for 4 h per day for 20 consecutive days), liver IL-1β and TLR4 mRNA levels were significantly increased, while NF-κB mRNA levels were significantly decreased in comparison to the control group birds kept in normal physiological conditions [199]. Contrary to the former, heat stress (32 ± 1 °C, 6 h/day for 9 weeks) in 25 week old Roman egg-laying hens was shown to be associated with increased serum inflammatory cytokine (IL-1β, IL-6, and TNF-α) response as compared to control nonstressed birds. Furthermore, heat stress was also responsible for significantly increased proliferating cell nuclear antigen (PCNA), TLR4, and NF-κB protein expression [200]. The authors showed the protective anti-inflammatory effects of curcumin (100 and 200 mg/kg) in the heat-stressed layers. Similarly, in black-boned chickens exposed to circular heat stress, dietary supplementation with resveratrol (400 mg per kg) was shown to improve intestinal integrity and ameliorate the mRNA overexpression of HSP70, HSP90, and NF-κB on the 6th, 10th, and 15th days of stress [201].

It seems likely that cold stress can also impose oxidative stress and enhance in vivo proinflammatory cytokine gene expression in chickens [202]. In fact, the expression of inflammatory factors (iNOS, COX-2, NF-κB, TNF-α, and prostaglandin E synthases (PTGEs) were shown to be increased in chicken heart due to cold stress [203]. Under cold stress in quail, the SOD activity decreased, reflecting an oxidative stress state, while the mRNA expression of NF-κB increased in the duodenum, jejunum, and ileum [204]. The inflammatory factors (COX-2, PTGEs, iNOS, NF-κB, and TNF-α) and Hsp70 mRNA levels were shown to be increased in quail spleen as a result of the acute and chronic cold stress (12 ± 1 °C) compared with birds in the control groups [205]. Increased malondialdehyde (MDA) content and upregulation in HSP27, HSP40, HSP70, NF-κB, COX-2, PTGEs, iNOS, TNF-α, and IL-4 mRNAs, as well as in protein levels of HSP40, NF-κB, and iNOS, were observed in heart due to acute cold stress (7 °C for 24 h) in broiler chickens [206]. Therefore, both heat and cold stress in poultry could be responsible for oxidative stress and inflammation, NF-κB proven to play crucial roles in the regulation of those processes.

### 6.2. Mycotoxins

Mycotoxins are considered as major nutritional stress factors in poultry production [1] imposing oxidative stress, immunosuppression [207], and low-grade inflammatory response in the chicken intestine [44] and compromising intestinal barrier functions [208]. Among feed-contaminating mycotoxins, AFB1 is considered to be the most toxic mycotoxin. A low level of AFB1 in broiler diet (74 μg/kg) was shown to increase the serum levels of MDA, TNF-α, and IFN-γ. These changes were inhibited by alpha-lipoic acid (α-LA) dietary supplementation (300 mg/kg). Interestingly, the activities of total SOD and GPx and the expression of NF-κB p65 and HO-1 were not affected by AFB1 [209]. In a similar experiment, an AFB1-contaminated diet (74 μg/kg) fed to chickens was associated with upregulation of the proinflammatory cytokine IL-6 and an increase in the protein expressions of both NF-κB p65 and i-NOS in the liver. Those negative effects of dietary AFB1 were shown to be inhibited by dietary alpha-lipoic acid (300 mg/kg [210]).

In an experiment with chicken feed contaminated with 1 mg/kg AFB1 fed from day 1 until day 28, broilers exposed to AFB1 were characterized by increased serum concentrations and mRNA expressions of TNF-α, IFN-γ, IL-1β, IL-10, and IL-6 as compared to the control group. In addition, AFB1 caused increased degradation of the IκBα protein and significantly elevated the phosphorylation of NF-κB (p65). Furthermore, AFB1 was responsible for a significant reduction in the mRNA level and protein expression of the Nrf2 gene. As a result, the mRNA expression and protein expression level of Nrf2-dependent antioxidant genes (HO-1, GPx1, NQO1, and GCLC) in the AFB1 group were shown to be significantly downregulated [211]. Interestingly, the authors demonstrated that most aforementioned changes in NF-κB and Nrf2-related parameters were partly alleviated by feeding grape seed proanthocyanidin extract (250 mg/kg) simultaneously with AFB1.

### 6.3. Mineral Dietary Excess and Heavy-Metal Contamination

#### 6.3.1. Mn, Cu, and NF-κB

Mn excess (600–1800 mg/kg feed) was shown to be associated with upregulated mRNA expression of TNF-α, COX-2, NF-κB, iNOS, and NO content in chicken testis on the 60th and 90th days [212]. The inflammatory response and the mitochondrial dynamics and apoptosis under Cu (300 mg/kg for 90 days) exposure in the heart of chickens were also investigated. It was shown that Cu exposure induced NF-κB-mediated pro-inflammatory cytokines, and the mitochondrial network was suggested to be considered as the cytosolic sensor responsible for the induction of NF-κB-mediated inflammatory responses under stress conditions [213].

In chickens, dietary Cu excess (220 and 330 mg of Cu/kg dry matter) was shown to increase the number and area of splenic corpuscles, as well as the ratio of cortex and medulla in the thymus and bursa of Fabricius. Furthermore, excessive Cu intake was associated with decreased AO defenses, indicated by the reduced activities of SOD, CAT, and GPx and increased content of MDA. There were also increased TNF-α, IL-1, and IL-1β concentrations, upregulated mRNA levels of TNF-α, IFN-γ, IL-1, IL-1β, IL-2, iNOS, COX-2, and NF-κB, and increased protein levels of TNF-α, IFN-γ, NF-κB, and p-NF-κB in immune organs due to Cu toxicity [214].

#### 6.3.2. As and NF-κB

The proinflammatory activities of As were shown in different tissues of birds, including liver, heart, brain, muscles, and kidney. For example, in birds chronically treated with As_2_O_3_, the expression levels of NF-κB and IL-6, IL-8, and TNF-α (critical mediators in the inflammatory response) in the liver were shown to be increased [215]. Indeed, As_2_O_3_ exposure (7.5–30 mg/kg for 90 days) led to oxidative stress, inflammatory response, and histological and ultrastructural damage, as reflected by altered levels of cardiac enzymes in chicken heart tissues. In addition, the messenger RNA levels of NF-κB and inflammatory cytokines (TNF-α, COX-2, NOS, and PTGEs) significantly increased due to As_2_O_3_ intoxication [216]. Similarly, when As_2_O_3_ (1.25 mg/kg body weight (BW), corresponding to 15 mg/kg feed) was added to a basal diet and fed to male Hy-line chickens (1 day old) for 4, 8, and 12 weeks, the expression of TNF-α, NF-κB, and iNOS in chicken heart was shown to be increased compared with the corresponding control group [217].

Arsenic (7.5, 15, or 30 mg/kg feed) was shown to increase the expression of NF-κB and proinflammatory cytokine expression in *Gallus gallus* brain tissues including cerebrum, cerebellum, thalamus, brainstem, and myelencephalon [218]. The toxic effects of arsenic trioxide (As_2_O_3_, 7.5–30 mg/kg for 30–90 days) in the muscular tissues (wing, thigh, and pectoral) of chickens were also investigated. The results showed that As_2_O_3_ caused oxidative stress as indicated by decreased activities of AO enzymes (catalase (CAT) and GPx) and increased MDA content. There was a significant upregulation of the mRNA levels of NF-κB and inflammatory cytokines (TNF-α, COX-2, iNOS, and PTGEs) and heat-shock proteins (HSPs) in muscular tissue in the As_2_O_3_ exposure groups [219]. In Hy-line chickens, As_2_O_3_ exposure (7.5, 15, and 30 mg/kg diet) was shown to induce oxidative stress and inflammatory-mediated nephrotoxicity. In fact, elevated nuclear migration of NF-κB and inflammation-related phenotypes were observed. They led to marked renal injury and apoptosis through a mitochondrion-dependent pathway in chicken kidneys [220].

#### 6.3.3. Cu, As, and NF-κB

Oxidative stress-induced skeletal muscle injury due to Cu^2+^ (300 mg/kg feed) and/or arsenite (2.5 mg/kg BW, corresponding 30 mg/kg feed) exposure in chickens was associated with inflammation in skeletal muscles induced via the NF-κB-mediated response pathway. Indeed, the increased protein and mRNA levels of NF-κB and TNF-α in skeletal muscles and the enhanced mRNA expressions of IL-1β, IL-6, and IL-12β were indicative of proinflammatory responses occurring due to Cu and/or As exposure [221]. Arsenic (30 mg/kg) and/or copper (300 mg/kg for 12 weeks) were shown to induce oxidative stress, inflammation, and autophagy in chicken brains. In fact, the mRNA levels and protein expressions of inflammation markers (NF-κB, TNF-α, COX-2, and PTGEs) were shown to be significantly increased due to As and Cu exposure [222]. Chicken exposure to As (30 mg/kg) and/or Cu (300 mg/kg for 4.8 and 12 weeks) was shown to lead to oxidative stress, inflammatory response (an increase in expression of NF-κB and its downstream inflammation-related genes), and liver damage through mitochondrial and death receptor-dependent pathways [223]. Arsenic trioxide (30 mg/kg) and/or copper sulfate (300 mg/kg) were added to the chicken basal diet for 12 weeks. Significantly reduced thymus weight and thymus index, hyperemia visible to the unaided eye, and inflammatory cell infiltration were observed. Concurrent administration of arsenic and copper significantly enhanced inflammation as indicated by increased levels of NF-κB, COX-2, iNOS, PTGEs, and proinflammatory cytokines in chicken thymus. Additionally, oxidative stress imposed by As and Cu was associated with elevation of the heat-shock protein levels [224].

Increased NF-κB expression and inflammation induction in chicken gizzard were also shown to be a result of As_2_O_3_ and/or CuSO_4_ dietary exposure [225]. Similarly, As and/or Cu exposure in the same doses was shown to induce immunotoxicity through triggering oxidative stress, inflammation (upregulation of NF-κB, inflammatory mediators, and proinflammation cytokines, accompanied by depletion of anti-inflammatory cytokines), and immune imbalance (decreased ratio of IFN-γ/IL-4 and increased level of IL-17) in the bursa of Fabricius of chicken [226]. In the chronic poisoning of Cu and/or As, inflammation occurs in the chicken thalamus as indicated by increased NF-κB expression, causing oxidative stress (MDA accumulation) and mitochondrial damage, leading to apoptosis [227]. Excessive intake of As (1.25 mg/kg BW) and/or Cu (CuSO4, 300 mg/kg feed) for 12 weeks was shown to lead to a significant reduction in the total antioxidant capacity (T-AOC), catalase level, and hydroxyl radical formation in chicken brain. In addition, an increase in the expression of HSPs and NF-κB, as well as NF-κB pathway-related proinflammatory mediators (COX-2, TNF-α, and iNOS), due to As/Cu intoxication was observed [228]. Therefore, the proinflammatory activities of Cu and As combinations were confirmed in the chicken liver, thymus, bursa of Fabricius, gizzard, thalamus, and brain.

#### 6.3.4. Pb and NF-κB

Pb poisoning in chickens was shown to increase the mRNA expression of inflammation factors (NF-κB) and HSPs in chicken livers simultaneously with the induction of NO content and iNOS activity [229]. It was shown that Pb exposure was associated with increased Pb content in chicken serum, induced the NF-κB pathway, and increased the expression of selenoproteins in chicken neutrophils [230].

More data on Pb-associated modulation of the expression of NF-κB and related cytokines is subsequently discussed in the Se section.

#### 6.3.5. Cd and NF-κB

It was shown that Cd significantly induced the expression of NF-κB, leading to activation of its downstream cytokines, IL-1β, TNF-α, and IL-6, in chicken peripheral blood lymphocytes [231]. As a result of CdCl_2_ (10 mg/kg feed) administration to chickens for 90 days, levels of NF-κB and phosphorylated c-Jun N-terminal kinase (p-JNK)/JNK in the spleen increased significantly, while those of mechanistic target of rapamycin (mTOR) and HSP70 decreased [232]. Exposure of 120 day old layers to Cd (150 mg/kg for 120 days) was associated with oxidative stress, increased NO production, iNOS activity, and increased expression of inflammatory factors (iNOS, NF-κB, TNF-α, and PTGE) and heat-shock proteins (HSPs 27, 40, 60, 70, and 90) in the liver tissues of birds [233]. In livers of duck exposed to a combination of molybdenum and cadmium, mRNA expression of Hsp60, Hsp70, Hsp90, TNF-α, NF-κB, and cyclooxygenase-2 (COX-2) was significantly upregulated [234]. Nickel chloride (NiCl_2_) was shown to cause inflammatory responses, indicated by activation of the NF-κB pathway and a reduction in the expression of anti-inflammatory mediators in broiler chicken kidney [235].

Cd exposure was associated with oxidative stress as indicated by increased MDA and reduced SOD and GPx in chicken peripheral blood lymphocytes. Interestingly, *Astragalus* polysaccharide was shown to inhibit Cd-induced cytotoxicity through regulation of NF-κB signaling [231]. It was shown that *Agaricus blazei* Murill polysaccharide (ABP) significantly reduced the accumulation of Cd in the chicken spleens and reduced the expression of NF-κB and its downstream inflammatory cytokines (IL-1β, IL-6, TNF-α, and IFN-β). Interestingly, ABP ameliorated the Cd-induced increase in protein levels of HSPs (HSP60, HSP70, and HSP90) in spleens. Furthermore, the activities of main antioxidant enzymes (SOD and GPx) significantly increased, while lipid peroxidation (MDA) decreased in the ABP + Cd group [236].

Therefore, as indicated by the above-presented data, the toxic effects of heavy metals (As, Pb, and Cd) and Cu excess in poultry have been associated with oxidative stress and increased expression and activity of NF-κB in various tissues, leading to inflammation. It seems likely that usage of various protective nutrients can prevent oxidative stress and control/decrease NF-κB expression. This can be demonstrated with plant polysaccharide or selenium (see Section 7.1) dietary supplementation.

### 6.4. Other Toxic Stress Factors

Hydrogen peroxide (H_2_O_2_) was shown to cause oxidative stress and impair redox status in farm animals [237] and poultry [238]; therefore, intraperitoneal injection of H_2_O_2_ can be used as an important model of oxidative stress in poultry.

Air quality, especially increased ammonia (NH_3_) and hydrogen sulfide (H_2_S) concentrations, is an important factor influencing poultry health and bird performance, including feed efficiency, growth rate, carcass quality, and susceptibility to diseases. Indeed, harmful concentrations of NH_3_ and H_2_S can suppress/dampen adaptive immune responses [239].

#### 6.4.1. H_2_O_2_

Oxidative stress in chickens induced by H_2_O_2_ injection was shown to suppress NF-κB signal activation and initiate autophagy in breast muscles [240]. In an experiment, Arbor Acres chickens were grown for 42 days, and, on days 16 and 37 of growth, control chickens were injected with saline, while experimental chickens received an intraperitoneal injection of H_2_O_2_ with 0.74, 1.48, and 2.96 mM/kg BW.

It was shown that the two highest doses of H_2_O_2_ imposed oxidative stress (decreased SOD and GPx activity), disturbed the redox balance, and significantly decreased the expression of NF-κB and its subunits (p50 and p65) in the chicken liver on day 42, triggering apoptosis and autophagy [241]. Indeed, H_2_O_2_ is considered to be a central redox signaling molecule in physiological conditions, while increased concentrations of H_2_O_2_ (>100 nM) can cause oxidative stress [242].

#### 6.4.2. NH_3_

Ammonia was shown to increase NF-κB expression in chicken trachea, associated with activation of downstream inflammation genes including iNOS and COX-2, reflecting a respiratory inflammation response [243]. The NH_3_-induced immunotoxic effects and inflammatory damage of broiler spleens were associated with the Th1/Th2 imbalance, NF-κB pathway, and compensatory response of HSPs. In particular, NH_3_ exposure led to inflammatory damage, indicated by decreased inflammation-related miRNAs (miR-133a and miR-6615), cytokines secreted by Th1 cells, and HO-1. Furthermore, the increased expression of two target genes of the two miRNAs, three cytokines secreted by Th2 cells, seven inflammation-related factors, and five heat-shock proteins was observed in broiler spleens due to NH_3_ exposure [244]. In a broiler model of ammonia exposure, it was shown that NH_3_ excess was associated with reduced breast weight and thigh weight, histopathological changes in kidney tissues, and increased iNOS activity and NO content. Furthermore, the mRNA and protein expression of inflammatory factors, including NF-κB, COX-2, prostaglandin E synthases, and iNOS, increased. At the same time, T helper 1 and regulatory T cytokines were shown to be downregulated with simultaneous upregulation of Th2 and Th17 cytokines [245]. A study was conducted to investigate NH_3_-induced inflammation in chicken bursa of Fabricius and thymus. Experimental chickens were divided into three groups: low (5.0 mg/m^3^), middle (10.0–15.0 mg/m^3^), and high (20.0–45.0 mg/m^3^) NH_3_-treated chickens. In comparison to the low NH_3_-treated group, high NH_3_ exposure was shown to induce inflammation associated with increased nuclear debris and vacuoles in the cortex and medulla of thymus and bursal follicles. Furthermore, reduced bursa of Fabricius and thymus index and increased NO content and iNOS activity due to high NH_3_ exposure for 14, 21, or 42 days were observed. Lastly, the inflammatory cytokine contents and mRNA levels of NF-κB, COX2, TNF-α, IL-6, IL-10, IL-1β, IL-18, TLR-2A, and iNOS were also increased in conditions of high NH_3_ exposure [246].

The effect of ammonia (1 mmol/L and 5 mmol/L) on chicken splenic lymphocyte apoptosis was studied. The results showed that NH_3_ exposure imposed oxidative stress, indicated by the increased release of calcium (Ca^2+^) and ROS from mitochondria. Furthermore, an increase in the mRNA levels of GPx, inflammation-related genes (NF-κB, COX-2, iNOS, TNF-α, and transforming growth factor-β (TGF-β)), and apoptosis-related genes (B-cell lymphoma 2, BCL-2; Bcl-2-associated X protein, BAX; cytochrome C, Caspase-9, and Caspase-3), and an increase in protein levels of NF-κB, iNOS, BAX, cytochrome C, Caspase-9, and Caspase-3 were also observed due to ammonia exposure. This was also associated with a decreased expression of GST and HO-1 in splenic lymphocytes exposed to ammonia [247]. In chickens, the spleen tissues were seriously injured due to high ammonia concentration (45 ppm from day 22 for 3 weeks) exposure. In the same group of birds, there was increased expression of IL-4, IL-6, and IFN-γ and decreased expression of IL-2 in the spleen, showing an imbalance in the Th1/Th2 response. Furthermore, the proinflammatory factors, including NF-κB, COX-2, iNOS, and prostaglandin E (PGE), were also upregulated in the high ammonia-exposed chickens [248].

#### 6.4.3. H_2_S

It is known that the decomposition of sulfur-containing organics in poultry houses is responsible for the production of a large amount of H_2_S, a highly toxic air pollutant, having detrimental effects on poultry health and leading to extensive damage to the body. In poultry, H_2_S exposure is thought to damage the respiratory system and cause an inflammatory reaction. In particular, it was shown that H_2_S exposure can inhibit the anti-inflammatory and antioxidant effects of PPAR-γ/HO-1 and activate proinflammatory NF-κB pathway-related genes and downstream genes, leading to aggravation of pneumonia induced by LPS. In particular, the expression of IL-4, IL-6, TNF-α, and IL-1β was increased and that of IFN-γ decreased, and the level of PPAR-γ/HO-1 was significantly suppressed by H_2_S exposure. Furthermore, the increased expression of I-κB-β and NF-κB genes confirmed that the NF-κB pathway was activated, with subsequent activation of COX-2, PGE, and iNOS [249].

Fourteen day old chickens were exposed to 30 ppm H_2_S for 14 days, and inflammation and oxidative stress indices were determined in the lymphocytes from peripheral blood samples. An increase in the inflammatory response associated with upregulation of the heat-shock proteins, NF-κB, COX-2, and iNOS was detected in the H_2_S group in comparison to the control untreated chickens [250]. Furthermore, H_2_S exposure (0–3 weeks: 4 ppm, 4–6 weeks: 20 ppm of H_2_S gas) was shown to induce oxidative stress and energy metabolism dysfunction. It also led to necroptosis, activated the MAPK pathway, and triggered the NF-κB pathway associated with a promotion of inflammatory response in chicken spleens [251]. To study the immunotoxicity of H_2_S, 1 day old broiler chicks were exposed to atmospheric H_2_S for 42 days. As a result, H_2_S was shown to activate the TLR-7/MyD88/NF-κB pathway and the NOD-like receptor protein 3 (NLRP3) inflammasome to promote an inflammatory response, leading to tissue damage in broiler thymus and a Th1/Th2 imbalance. In fact, H_2_S was indicated to significantly induce IL-1β, IL-4, and IL-10 levels, and it downregulated IL-12 and IFN-γ. In addition, mitochondria were shown to be swollen, the chromatin was condensed, and nuclear structures were destroyed due to H_2_S exposure [252].

### 6.5. LPS-Induced Stress

The stimulating effect of LPS on NF-κB expression was shown in vitro in model systems and in vivo with poultry. For example, chicken thrombocytes responded to LPS through TLR4, MAP kinase, and NF-κB pathways associated with increased expression of IL-6 and cyclooxygenase-2 and enhanced production of prostaglandin E2 [253]. Similarly, in chicken thrombocytes, LPS-induced IL-6 production was shown to be mediated via activation of NF-κB, extracellular-signal-regulated kinase (ERK) ½, and MAPK [254]. Furthermore, LPS was shown to upregulate IL-6 and CXCLi2 gene expression in chicken heterophils via ERK1/2-dependent activation of AP-1 and NF-κB signaling pathways [255]. In laying hens, NF-κB was shown to participate in the induction of mucin expression by LPS in the vaginal mucosa, improving barrier function against infections [256]. The LPS challenge led to an increased mRNA abundance of TLR4, NF-κB, IL-1β, and IL-6 jejunal mucosa of broilers. However, these effects of the LPS administration were ameliorated by dietary *Astragalus* polysaccharide [257]. *Salmonella* LPS injection was found to induce liver damage as indicated by increased necrotic symptoms, severe fatty degeneration, increased alanine aminotransferase (ALT) activity, ballooning degeneration, congestion, and increased inflammatory cell infiltration in liver sinusoids. Significant upregulation in TLR4 expression and its downstream molecules (e.g., NF-κB, MyD88, TNF-α, IL-1β, and TGF-β), increased apoptosis, and decreased proliferation were also observed [258]. Acute spleen injury induced by LPS in young chicks was shown to be associated with significant upregulation of TLR4 at 36 h post LPS stimulation and a slight increase in the expression of NF-κB at 12 h post LPS treatment. The NF-κB-regulated cytokines (TNF-α and IL-6) were shown also to exhibit significant upregulation at 12 h following LPS stimulation [259]. The aforementioned data clearly indicate that LPS can activate NF-κB expression in vitro and in vivo.

An LPS-induced ileum injury model in chickens was established, and histological examination showed a fragmented structure of blood vessels in the ileum and presence of necrotic tissue in the lumen in the LPS-treated chickens. In the LPS group, the structure of the villi was chaotic with rough and uneven surface [238]. Moreover, in comparison to the control group, LPS (60 mg/kg) induced an increase in TLR4 protein expression levels and p65/p65 ratio, increased the mRNA expression of IL-6, IL-8, and TNF-α, and decreased the mRNA expression of IL-10 [260]. Dihydromyricetin (DHM), a natural flavonoid compound with anti-inflammatory activity (0.05% and 0.1%), was shown to have protective effects against LPS-induced inflammatory responses, including regulation of NF-κB expression [260]. Supplementation with leonurine hydrochloride (LH), an alkaloid isolated from *Herba leonuri*, attenuated LPS-induced intestinal inflammation and barrier dysfunction by significantly downregulating the mRNA expression of NF-κB, COX-2, and proinflammatory cytokines (TNF-α, IL-1β, and IL-6) in the jejunal mucosa. Furthermore, LH administration attenuated LPS-induced IκBα phosphorylation and nuclear translocation of NF-κB (p65) in the jejunal mucosa [261].

### 6.6. Diseases

Modern breeds/strains of commercially grown meat-type broiler chickens are characterized by increased body weight, improved meat yield, including the *Pectoralis major* (breast) muscle, improved feed conversion, and decreased time to processing. However, myopathies affecting meat quality, especially in the *Pectoralis major* muscle, are considered as a major challenge for modern broiler production. It seems likely that broiler breast muscle myopathies are associated with inflammation [262]. The NF-κB signaling pathway was found to be induced, the mRNA expression levels of downstream inflammatory mediators were increased, and TLR levels were upregulated in *Pectoralis major* of Wooden breast myopathy-affected broiler chickens [263]. The authors also showed that, in the serum of broilers with breast myopathies, contents of IL-1β, IL-8, and TNF-α were increased. At the same time, in breast muscle, the mRNA expression of inflammatory cytokines was dysregulated, showing association of this myopathy with an immune disorder and systemic inflammation response.

The regulatory roles of NF-κB in the development and pathogenesis of various bacterial and virus diseases were recently studied. In particular, the roles of NF-κB and inflammation in the pathogenesis of pathogenic *Escherichia coli*, various *Salmonella* species, and *Mycoplasma gallisepticum,* *Eimeria tenella* and *Clostridium perfringens* have received the most attention among bacterial diseases. Infectious bursal disease and Newcastle disease were on the frontline for understanding roles of inflammation and NF-κB in their pathology.

#### 6.6.1. *Escherichia coli*

*Escherichia coli* is known to be a Gram-negative, facultative anaerobe bacterium belonging to the Enterobacteriaceae family [264]. Certain *E. coli* strains, known as “avian pathogenic *E. coli*” (APEC) are responsible for colibacillosis, one of the most important causes of chicken mortality in the poultry industry worldwide [265]. To explore the host–pathogen interaction, a response in global gene expression profiling of chicken type II pneumocytes (CP II cells), responsible for secreting surfactants and modulating lung immunity, to avian pathogenic *Escherichia coli* (APEC-O78) infection was determined. In fact, CP II cells were shown to respond to APEC infection with marked changes in the expression of 1390 genes (from 18,996 genes identified) with 803 downregulated mRNAs and 587 upregulated mRNAs [266]. The major enriched pathways were identified to be related to the NF-κB signaling pathway, apoptosis pathway, tight junction, and cytokine–cytokine receptor interaction. Furthermore, the top 15 upregulated biological process terms were found to include regulation of the Toll signaling pathway, apoptotic process, and intracellular signal transduction [244]. The expression of phosphorylated NF-κB p65 and phosphorylated IκB was significantly upregulated in the APEC-infected chicken type II pneumocytes compared with the control group. However, baicalin, a medicinal ingredient isolated from dry roots of *Scutellaria baicalensis* Georgi, was shown to significantly inhibit the expression of phosphorylated NF-κB p65 and phosphorylated IκB induced by APEC-O78 [267].

Furthermore, the protective effects of baicalin on avian pathogenic APEC-induced acute lung injury associated with NF-κB activation and inflammation in chicken were shown [268]. Artemisinin, a drug derived from the Asian plant *Artemisia annua*, was shown to alleviate *Eimeria tenella* infection in chickens as a result of facilitating the apoptosis of host cells and suppressing the inflammatory response by suppressing the increased mRNA expressions of NF-κB and interleukin-17A in ceca during infection [269]. Schizandrin, a group of bioactive chemical compounds found in *Schisandra chinensis,* was shown to attenuate inflammation induced by APEC-O78 in chicken type II pneumocytes by decreasing the levels of IL-1β, IL-8, IL-6, and TNF-α via its inhibitory effect on NF-κB and MAPK activation [270]. Dietary treatment with both live yeast and mannan oligosaccharide was shown to alleviate *E. coli*-induced increases in ileal Toll-like receptor 4, NF-κB, and IL-1 β expression in broilers [271].

#### 6.6.2. *Salmonella*

*Salmonella*, a Gram-negative bacterium belonging to the Enterobacteriaceae family, is commonly found in the digestive tract of infected chickens. Furthermore, it is an important cause of foodborne human illnesses worldwide, and poultry meat is reported to be responsible for up to 25% of outbreaks caused by foodborne pathogens [272]. Infection of chicken TLR5 transfected cells with *Salmonella enterica* serovar Enteritidis was shown to activate NF-κB in a dose- and flagellin-dependent fashion [273]. In order to study the role of NF-κB in the signal transduction pathway of the *Salmonella enteritidis*-challenged cells, chicken macrophage HD11 cell line and small interfering RNAs (siRNA), specifically inhibiting NF-κB1 expression, were used. In particular, it was found that a 36% inhibition of NF-κB1 expression was associated with increased gene expression of both TLR4 and IL-6 at both 1 h and 4 h following *Salmonella* challenge [274]. TLR4 was shown to activate NF-κB signaling during cerebral ischemia–reperfusion, leading to increased secretion of inflammatory cytokines and damage of brain tissue [275]. Nucleotide-binding oligomerization domain-containing protein-1 (NOD1), known as a cytoplasmic pattern recognition receptor (PRR), is considered to be a key member of the NOD-like receptor (NLR) family. As a result of recognition of various pathogens by NLRs, NF-κB signaling is modulated, leading to induction of the host innate immune response. In fact, following *S. enterica* serovar Enteritidis infection, induced expression of chicken NOD1 and NF-κB was demonstrated [186]. In carrier chickens challenged with *Salmonella enterica* serovar Pullorum, upregulation of NF-κB and NRLC5 signaling pathways at different persistence periods was observed [276].

The *Salmonella* secreted factor L, a deubiquitinase that contributes to the virulence of *Salmonella* Typhimurium, was shown to suppress the intracellular NF-κB pathway associated with enhancement of the virulence of *Salmonella* Pullorum in a chicken model [277]. In chickens, *Salmonella* Typhimurium was shown to significantly reduce chicken performance, including the feed intake and body weight gain, detrimentally affecting the feed conversion ratio. At the same time, *Salmonella* infection induced the inflammatory expressions of NF-κB and MyD88 genes and decreased the expressions of claudin-1, occludin, and mucin-2 tight junction genes in the intestines. Furthermore, *S.* Typhimurium was reported to significantly decrease ileal bacterial diversity indices [278,279]. The invasion plasmid antigen J (IpaJ) from *Salmonella* Pullorum was reported to suppress NF-κB activation by inhibiting IκBα ubiquitination and modulating the subsequent inflammatory response [280].

#### 6.6.3. *Mycoplasma gallisepticum*

*Mycoplasma gallisepticum* (MG), an avian pathogen, belonging to the class of Mollicutes, is known as the primary etiological agent of chicken chronic respiratory disease, causing inflammatory damage of the host respiratory system [281]. Initially, when live MG bacteria were incubated with primary chicken tracheal epithelial cells, inflammatory NF-κB-dependent genes were upregulated, while an NF-κB inhibitor abrogated the inflammatory response [282]. Furthermore, TLR2-2 and TLR6 were reported to be upregulated upon MG infection, followed by induction of the NF-κB-mediated inflammatory responses [283]. At the next stage of the research related to the relationship between MG infection and NF-κB expression, microRNas were employed. Indeed, noncoding RNAs, including microRNAs (miRNAs), are known to be involved in the regulation of various cellular processes including gene expression at the post-transcriptional level [284]. Among them, MiR-21 is an evolutionarily conserved miRNA found in a wide range of vertebrate species, including mammals and birds [285].

Recently, it was shown that, in order to provide an effective defense against MG infection, gga-miR-21 is involved in the activation of MAPKs and NF-κB signaling pathways, leading to increased production of inflammatory cytokines and suppressing cell apoptosis [286]. Similarly, upon MG infection, gga-miR-146c upregulation was shown to repress MMP16 expression and activate the TLR6/MyD88/NF-κB pathway. This was associated with inhibiting cell apoptosis and promoting cell proliferation, important events to defend against host MG infection [287]. Furthermore, upregulating gga-miR-16-5p was reported to decrease multiplication and cycle progression and increase apoptosis of *MG*-infected DF-1 cells, by inhibiting the phosphatidylinositol 3-kinase (PI3K)/protein kinase B (Akt)/NF-κB pathway to exert an anti-inflammatory effect [288]. In a model system with DF-1 cells in chicken embryo fibroblasts, gga-miR-146c was shown to activate the TLR6/MyD88/NF-κB pathway through targeting matrix metalloproteinase-16 (MMP16) to prevent MG infection in chickens [287]. Upon MG infection, upregulation of miR-130b-3p was shown to activate the PI3K/Akt/NF-κB pathway and induce cell proliferation as a result of downregulating phosphatase and tensin homolog (PTEN). Importantly, inhibition of miR-130b-3p led to the opposite results [289]. In DF-1 cells exposed to *Mycoplasma* *gallisepticum*, lipid-associated membrane proteins were reported to induce IL-1β production through the NF-κB pathway [290]. Indeed, MG infection was shown to trigger an inflammatory response through the TLR-2/MyD88/NF-κB signaling pathway, leading to tissue damage in chicken thymus [43,291].

Polydatin (PD), a resveratrol glycoside isolated from *Polygonum cuspidatum*, with prominent anti-inflammatory activity, was used as a therapeutic means against MG-induced inflammation in chickens. First, histopathological studies clearly showed that PD treatment (15, 30, and 45 mg/kg) was able to alleviate MG-induced pathological changes in the chicken embryonic lung. Second, PD treatment (15, 30, and 60 μg/mL) was shown to significantly suppress the expression of IL-6, IL-1β, and TNF-α induced by MG in chicken embryo fibroblast (DF-1) cells. Furthermore, the MG-induced levels of TLR6, MyD88, and NF-κB were also significantly decreased by PD treatment, which restrained the MG-induced NF-κB-p65 nuclear translocation [292].

As mentioned above, MG can target host cells and cause chronic respiratory disease in chicken. In fact, in chicken spleen and DF-1 cells, MD infection was shown to impose oxidative stress and inflammation. However, baicalin was reported to suppress TLR2–NF-κB signaling pathway by inhibiting the phosphorylation of p65 and IκB [293]. Interestingly, baicalin was reported to restore the mRNA expression of mitochondrial dynamics-related genes and maintain the balance between mitochondrial inner and outer membranes, as well as upregulate the Nrf2/HO-1 pathway and suppress the NF-κB pathway in the spleen of MG-infected chicken [294]. Similar protective effects of baicalin [295] and polydatin, a resveratrol glycoside isolated from *Polygonum cuspidatum* [292], against MG-induced inflammation injury in chicken embryonic lung were associated with their inhibition of the TLR6/MyD88/NF-κB pathway.

Puerarin (PUE), an isoflavone found in a number of plants and herbs, was shown to inhibit MG-induced inflammation and apoptosis via suppressing the TLR6/MyD88/NF-κB signal pathway in chickens. In fact, compared to the MG-infected group, PUE was found to effectively inhibit the expression of MG-induced inflammatory genes, including TNF-α, IL-1β, IL-6, TLR6, MyD88, and NF-κB. In particular, PUE was reported to dose-dependently inhibit MG-induced NF-κB p65 translocation to the cell nucleus [296].

#### 6.6.4. *Eimeria tenella*

Chicken coccidiosis is an enteric disease caused by *Eimeria* infection, leading to severe economic losses associated with immunosuppression and a high level of mortality in the poultry industry worldwide [297]. In fact, *Eimeria tenella* infection was shown to significantly increase the expression of NF-κB mRNA in chicken cecal tissue in vivo [269], and a similar increase in the expression level of NF-κB was observed in chicken intestinal epithelial cells in vitro after infection with *E. tenella* sporozoites [298].

There is a need for more research related to the regulatory roles of NF-κB pathway in the development of a chicken coccidiosis prevention strategy.

#### 6.6.5. *Clostridium perfringens*

*Clostridium perfringens*-induced necrotic enteritis in chickens has become an economically significant problem for the broiler industry [299,300], especially at farms that have stopped the use of antibiotic growth promoters [301]. It is known that the *Clostridium perfringens* main cell-wall component, peptidoglycan, can be recognized by TLR2 with subsequent activation of the NF-κB signaling pathway to induce cytokine and chemokine production, leading to inflammation [302]. The authors conducted an in vitro study with primary intestinal epithelial cells to assess the chicken intestinal inflammatory responses to *C. perfringens* and showed increased cytokine expression related to NF-κB activation [302]. Furthermore, pathways affected by the infusion of *C. perfringens* culture supernatant in the duodenum of broilers included NF-κB signaling, death receptor signaling, and an inflammatory response [303].

Importantly, two *Lactobacillus* species were shown to reduce the growth of *Clostridium perfringens* and inhibit the upregulation of NF-κB p65 in *C. perfringens*-challenged chicken intestinal epithelial cells [304]. Indeed, inclusion of *L. acidophilus* into the chicken diet was shown to improve gut health and reduce the mortality of *Clostridium*-challenged broiler chicks suffering from necrotic enteritis [305].

#### 6.6.6. *Chlamydia psittaci*

*Chlamydia psittaci*, a pathogen in poultry and pet birds, is known to have some protective mechanisms to cope with proinflammatory mediators during the early host response, leading to effective evasion and causing psittacosis/ornithosis [306]. The polymorphic membrane protein D (PmpD) is known as a highly conserved outer-membrane protein helping the pathogen to decrease immune system protection during *Chlamydia psittaci* infection. Therefore, the ability of the N-terminus of PmpD (PmpD-N) to regulate the functions of chicken macrophages was studied. In particular, it was shown that stimulation of HD11 macrophages with PmpD-N was associated with an increased secretion of the Th2 cytokines, IL-6, and IL-10 and upregulated expression of TLR2, TLR4, MyD88, and NF-κB. In great contrast, inhibition of TLR2, MyD88, and NF-κB in HD11 cells was reported to significantly decrease IL-6 and IL-10 cytokine levels associated with significantly enhanced NO production and phagocytosis [307]. The plasmid-encoded protein CPSIT_P7 of *Chlamydia psittaci* was shown to induce the TLR4/Mal/MyD88/NF-κB signaling axis and orchestrate the inflammatory cytokine response [308].

It is important to mention that, during host cell infection, NF-κB is activated by various pathogens, leading to the creation of a hostile environment for invading infectious agents; however, the pathogen can diminish the protective inflammatory response by blocking NF-κB translocation to the nucleus [309].

#### 6.6.7. Infectious Bursal Disease

As mentioned above, NF-κB is involved in the pathogenesis of various virus-induced diseases. In fact, infectious bursal disease virus (IBDV) is known as the etiological agent of a highly transmissive and immunosuppressive disease detrimentally affecting domestic chickens (*Gallus gallus*) in commercial poultry production. Indeed, IBD (Gumboro disease) can cause high morbidity and mortality of infected birds, leading to major economic losses in the poultry industry worldwide. The danger of IBD is associated with its immunosuppressive action associated with a loss of IgM-bearing B lymphocytes and the destruction of the bursa of Fabricius [72]. There is some evidence indicating that IBDV infection can cause oxidative stress in chickens [310], but regulatory roles of the antioxidant defense network in IBD need further elucidation.

IBDV infection was found to induce spleen macrophage activation via p38 MAPK and NF-κB pathways [311]. However, the molecular mechanisms of IBDV development and pathogenicity are still poorly understood; nevertheless, poorly regulated cytokine production and B-cell depletion due to apoptosis are believed to be important contributing factors to the disease pathology and severity. In IBDV-infected chicken embryonic fibroblasts, a great number of target genes and inducers of NF-κB were reported to be upregulated, in comparison to noninfected cells. It could well be that IBDV may support its replication and facilitate viral spread by affecting host-cell survival and apoptosis through NF-κB activation [312]. Interestingly, exacerbated apoptosis of cells infected with IBD virus upon exposure to IFN-α was shown to be associated with double-stranded RNA-dependent protein kinase (PKR), TNF-α, and NF-κB expression. Indeed, their downregulation is reported to drastically reduce the extent of apoptosis [313]. Protocatechuic acid (PCA), a type of widely distributed naturally occurring phenolic acid, was found to activate NF-κB signal pathways in the early stage of IBDV infection, leading to apoptosis promotion [314].

#### 6.6.8. Newcastle Disease

Newcastle disease (ND) is regarded as one of the most important avian diseases significantly affecting poultry production all over the world, being a great threat to the poultry industry [315]. It is well known that ND epidemics can cause high chicken mortality with great economic losses [315]. There is no effective treatment for the disease, and poultry producers rely on vaccination and strict biosecurity as vital measures for controlling the spread of the disease [316]. It is known that NDV can cause oxidative stress in poultry [310]; however, the roles of redox homeostasis in ND development are poorly understood. In fact, intense inflammatory responses leading to excessive cellular apoptosis and tissue damage were shown to be a result of Newcastle disease virus (NDV) infection in poultry. However, the molecular mechanisms of such actions have not been fully elucidated [317]. In NDV-infected chickens, glucocorticoid dexamethasone was shown to modulate NF-κB-dependent gene expression by upregulating FK506-binding protein 51 expression [318]. Furthermore, Newcastle disease virus-like particles were shown to induce dendritic cell maturation with synthesis of proinflammatory cytokines through the TLR4/NF-κB pathway [319]. Recently, it was shown that, during NFV infection, high-mobility group box 1 (HMGB1), a key member of the damage-associated molecular patterns (DAMPs), was responsible for NF-κB induction and a drastic increase in proinflammatory cytokine production in DF-1 and A549 cells [317]. It seems likely that activated NF-κB signaling can suppress NDV replication. This was shown experimentally with DF-1 cells (e.g., a chicken embryo fibroblast cell line) by using gga-miR-19b-3p, which enhanced NF-κB activity and led to increased inflammatory cytokine production and inhibition of NDV replication [320].

Expression of IFIT5 (interferon-induced protein with tetratricopeptide repeats 5) possessing antiviral activity and enhancing innate immunity was studied in chickens. The relative mRNA expression level of chicken IFIT5 (chIFIT5) was shown to be the highest in spleen, and the expression level of chIFIT5 was found to be significantly upregulated following NDV infection. In particular, it was shown that overexpression of chIFIT5 could promote IRF7- and NF-κB-mediated gene expression following NDV infection [321]. The DNA-sensing pathway is known to induce innate immune responses against DNA virus infection, with NF-κB signaling being critical for the establishment of innate immunity.

#### 6.6.9. Other Viral Diseases

It seems likely that Marek disease virus (MDV) and reovirus infections also affect NF-κB signaling. In fact, Marek’s disease (MD) is a neoplastic virus disease infecting chickens and frequently causing cancers in animals [322]. It was shown that NF-κB is involved in MDV-induced neoplastic transformation of CD30-expressing chicken lymphocytes in vivo [323]. Furthermore, chicken MD virus RLORF4 (a MDV-specific gene) was shown to inhibit type I interferon production by antagonizing NF-κB activation. In fact, RLORF4 binds to the Rel homology domains of the NF-κB subunits p65 and p50, interrupting their translocation to the nuclei and, thus, inhibiting IFN-β production [324].

Avian reoviruses are important pathogens causing infectious arthritis/tenosynovitis, stunting syndrome, respiratory disease and enteric disease, immunosuppression, and malabsorption syndrome in poultry [325,326]. Thus, avian reovirus can cause oxidative stress and disturb redox homeostasis in poultry [310]. Avian reovirus S1133 in cell cultures, in the early stages of infection, was shown to induce Akt/NF-κB and STAT3 signaling, leading to an inflammatory response and delayed apoptosis [327]. Furthermore, in avian reovirus-infected chickens, the expression peak for NF-κB in peripheral blood lymphocytes was shown to occur at 3 days post infection (dpi). Similarly, IFN-α, IFN-β, and IL-12 expression levels also peaked at 3 dpi, while IFN-γ, IL-6, IL-17, and IL-18 expression reached a maximum level earlier (at 1 dpi), whereas IL-8 (5 dpi) and IL-1β and TNF-α (7 dpi) peaked later [328]. Recently, the phosphoproteomic responses of duck to reovirus infections in the spleen tissue were studied, and 16 proteins involved the intracellular signaling pathways of PRRs were shown to be phosphorylated proteins. In particular, changes in the phosphorylation levels of NF-κB, as well as MyD88, receptor interacting protein 1 (RIP1), MDA5, and IRF7, indicated an important role of protein phosphorylation in duck immune responses to viral antigens [329]. Pattern recognition receptor signaling during innate responses to influenza A viruses in the mallard duck was recently reviewed [330], and the fundamental roles of NF-κB in innate immune responses to duck Tembusu virus infection were discussed in detail [331].

As can be seen from the above-presented data, NF-κB plays a pivotal role in poultry protection against major microbial and viral diseases, by regulating immunity and inflammation; however, the molecular mechanisms of its regulation in avian species await further investigation.

## 7. Nutritional Modulation of NF-κB in Poultry

### 7.1. Selenium

There is a great body of evidence indicating that the micronutrient selenium (Se) and selenoproteins are involved in the regulation of inflammatory signaling pathways, including NF-κB signaling, implicated in the pathogenesis of various diseases [13,332]. As a part of 25 selenoproteins, Se is involved in antioxidant defenses and the maintenance of redox balance [13].

The literature data related to the effect of Se on NF-κB and inflammation can be divided into three groups. Firstly, the detrimental effects of Se deficiency or excess on NF-κB signaling were shown. Secondly, the protective effects of Se in Pb and Cd toxicity were described. Thirdly, in LPS-induced models of oxidative stress and inflammation, Se was shown to be protective.

#### 7.1.1. Se Deficiency

Se deficiency in chickens was shown to lead to activation of the NF-κB pathway, with a change in selenoprotein gene expression resulting in kidney dysfunction [333]. Furthermore, Se deficiency was reported to attenuate chicken duodenal mucosal immunity via activation of the NF-κB signaling pathway. In particular, Se deficiency enhanced the phosphorylation of IκB-α and phosphorylation of kappa-B kinase subunit alpha (IKKα), as well as increased p50 and p65 DNA-binding activities. Furthermore, in Se deficiency, IKKα was elevated, but IκB-α was decreased [334]. The increasing levels of ROS in chicken duodenal mucosa due to Se deficiency could trigger NF-κB signal transduction [335]. In a recent experiment, the control group was fed a complete formula feed (0.2 mg Se/kg), while the experimental group of chickens was fed a self-made low-Se diet (0.004 mg/kg) for 15, 25, 35, 45, and 55 days. In chicken spleen at 15–45 days, the relative expression of TLR4 mRNA was shown to be increased due to Se deficiency. The relative expression of NF-κB mRNA in the experimental group was also increased in comparison to that in the control group at 15–45 days. The relative expression of IL-6 mRNA and the protein expression level of TLR4 in the experimental group were increased due to Se deficiency at 15–45 days of age [336]. The authors concluded that Se deficiency is associated with inflammatory injury as a result of the TLR4/TIR-domain-containing adapter-inducing interferon-β (TRIF)/NF-κB signaling pathway activation in chicken spleen.

Interestingly, the adverse effects of Se excess/toxicity (15 mg/kg Se for 45 days) on inflammatory and immune responses in chicken spleens were also associated with enhancement of the expression of NF-κB, iNOS, COX-2, PTGE, IL-6, TNF-α, and IL-4, but a depression of FOXP3 and IFN-γ [337]. However, Se dietary supplementation at 2 mg/kg did not affect the mRNA levels of NF-κB, COX-2, PTGEs, and TNF-α in chicken kidneys.

#### 7.1.2. Se and Pb Toxicity

Dietary Se has been proven to alleviate the Pb-induced increase in NF-κB and HSP expression in chicken livers [229]. Importantly, Se supplementation (1 mg/kg diet) was shown to reduce Pb concentration in serum, partly mitigated the effect on the activation of the NF-κB pathway, and further enhanced selenoprotein expression induced by Pb exposure [230]. One week old male chickens were treated via drinking water with Pb (350 mg/L) and provided with dietary Se (1 mg/kg) or both Pb and Se. On the 4th, 8th, and 12th weeks, kidneys were used to assess oxidative stress indicators, relative expressions of cytokines, and other inflammatory factors. The results showed that Pb consumption imposed renal injuries associated with increased lipid peroxidation (MDA), as well as the content and expression of IL-1β, IL-6, IL-17, NLRP3, caspase-1, NF-κB, COX-2, TNF-α, and PTGEs, and with reduced GSH content, as well as GPx and SOD activities, in the chicken kidneys. Se administration was shown to alleviate the aforementioned changes [338].

Pb treatment (50 mg/kg for 90 days) was shown to compromise AO defenses by inhibiting the activities of SOD, GPx, and CAT, causing the accumulation of NO and MDA, leading to oxidative stress, which promoted the expression of MAPK/NF-κB pathway genes (ERK, JNK, P38, NF-κB, and TNF-α) and activated HSPs (HSP27, HSP40, HSP60, HSP70, and HSP90) in chicken spleen. However, Pb-caused necroptosis was inhibited by Se (2 mg/kg) co-treatment [339]. Selenium (sodium selenite, 1 μM) was shown to prevent lead (30 μM)-induced necroptosis by restoring antioxidant functions (SOD, GPx, CAT) and blocking the MAPK/NF-κB pathway (decreasing the expression of NF-κB and TNF-α) in chicken lymphocytes [340].

#### 7.1.3. Se and Cd Toxicity

Dietary Cd (150 mg/kg for 90 days) was shown to increase the mRNA levels of NF-κB, COX-2, PTGEs, and TNF-α in chicken kidney. Interestingly, Se partly ameliorated the proinflammatory effects of Cd dietary supplementation [341]. Similarly, treatment with Se (2 mg/kg for 90 days) significantly alleviated Cd-induced hepatic toxicity (150 mg/kg) in laying hens, as evidenced by a reduction in Hsp60, Hsp70, Hsp90, NF-κB, COX-2, PTGEs, TNF-α, and IL-1β expression [342].

Chicken Cd exposure (150 mg/kg) was shown to activate inflammation-related genes including TNF-α, NF-κB, iNOS, COX-2, and prostaglandin E synthase (PTGEs) in chicken breast muscles [323,343]. Interestingly, Se (2 mg/kg as sodium selenite for 90 days) was reported to alleviate Cd-induced inflammation and meat quality deterioration via antioxidant and anti-inflammation action [343]. Supplementation with Se-yeast (0.5 mg/kg) was shown to have an antagonistic effect on Cd-induced inflammatory injury in chicken livers [233].

#### 7.1.4. Se and LPS

By inhibiting the phosphorylation of NF-κB, Se was shown to reduce breast tissue inflammatory injury induced by LPS [344]. In laying hens, LPS stimulation (injected LPS into the abdominal cavity at the age of 8 months) imposed oxidative stress, indicated by decreased activity of SOD, GPx, and CAT, decreased GSH content, and increased H_2_O_2_ and MDA content in the chicken myocardium. LPS also increased the expression of p38 MAPK and NF-κB, as well as TNF-α, IL-1, PTGE, COX-2, and iNOS. Interestingly, the addition of dietary SeMet (0.5 mg/kg for 4 months) was found to alleviate the changes in the above inflammation indicators [345]. Similarly, SeMet (0.5 mg/kg) was shown to inhibit the LPS-induced inflammation of liver tissue via suppressing the TLR4–NF-κB–NLRP3 signaling pathway in chickens [346].

In a recent experiment with 46 week old ISA laying hens, birds were injected with LPS (200 mg/kg) intraperitoneally, and, after 5 h, the tracheal tissue was collected for various assays. In the LPS-treated group, the epithelial cells were shown to be degenerated with necrotic changes accompanied by inflammation. The expression of the NF-κB pathway and related inflammatory factors, including TNF-α, iNOS, NF-κB, COX-2, and PTGEs, was significantly increased in the trachea tissue due to LPS treatment. In such conditions, increased (from 0.2 up to 0.5 mg/kg) SeMet supplementation for 90 days showed anti-inflammatory effects [347].

### 7.2. Amino Acids

Dietary l-arginine supplementation (1.05–1.9%) was shown to attenuate the LPS-induced inflammatory response in broiler chickens, as evidenced by the decreased expression of IL-1β, TLR4, and PPAR-γ mRNA in the spleen, and IL-1β, IL-10, TLR4, and NF-κB mRNA in the cecal tonsils [348]. There were no significant interactions between immune stress caused by bovine serum albumin (BSA) and supplementation of threonine (0.49–0.76% for 21 days) for NF-κB gene expression in the jejunum or ileum of Pekin ducks [349].

Interestingly, NF-κB expression in the jejunum was twofold higher than that in the ileum. Leucine was reported to alleviate LPS-induced inflammatory responses as a result of downregulating the NF-κB signaling pathway. In particular, a model system employing the intestinal tissue from specific pathogen-free chick embryos cultured in the presence of LPS for 2 h was used. LPS was shown to increase the phosphorylation of NF-κB while decreasing the phosphorylation level of mTOR. In this system, leucine supplementation at 40 mM was reported to suppress the phosphorylation levels of NF-κB, while restoring the phosphorylation level of mTOR [350].

### 7.3. Phytogenic Supplements

Recently, various phytogenic supplements received tremendous attention in poultry and animal nutrition, and the molecular mechanisms of their protective actions in many cases were related to their antioxidant properties. However, our analysis of the current data in this area showed that polyphenolic compounds are poorly absorbed and their concentrations in target tissues are several orders lower than those used in in vitro studies [351]. Furthermore, their antioxidant properties are condition-dependent, and, in many cases, polyphenols could show pro-oxidant activities. Therefore, it was suggested that the polyphenolic effects on NF-κB and Nrf2 expression could be a major molecular mechanism of their protective action in various model systems and in poultry nutrition in general [351,352]. Data presented in Section 4 showing the activation effects of polyphenol compounds on Nrf2 expression and activity with simultaneous suppression of the NF-κB pathway confirmed that idea. There are also a range of publications showing protective effects of phytogenic supplements in poultry nutrition under various stress conditions.

In chickens receiving conventional vaccinations, the NF-κB gene mRNA relative expression in hepatocytes linearly decreased as a result of increasing resveratrol, a plant-derived polyphenolic compound, with a dietary concentration from 200 to 800 mg/kg of diet [353]. Similarly, dietary resveratrol (200–600 mg/kg) was shown to reduce the protein expression of NF-κB, HSP70, and HSP90 in the jejunal chicken villi after 15 days of heat stress [201]. Dietary resveratrol (400 mg/kg) was also shown to protect quail hepatocytes against heat stress by decreasing the expression of NF-κB, Hsp70, and Hsp90, and increasing the hepatic and SOD, CAT, and GSH-Px activities [354]. Daidzein (DA), a soy isoflavone, included into the breeder diet at 20 mg/kg was shown to activate the NF-κB, MAPK, and Toll-like receptor signaling pathways of the offspring broilers. Furthermore, DA promoted lymphocyte development and differentiation and downregulated the expression of genes regulating lymphocyte apoptosis. It also increased the proportion of B cells, leading to promotion of Ig secretion with increased serum IgA and IgG levels and serum ND virus antibody titers [355]. In healthy Arbor Acre broilers, quercetin supplementation (0.04% and 0.06% for 6 weeks) was shown to significantly increase the expression of TNF-α, TNF receptor associated factor-2 (TRAF-2), TNF receptor superfamily member 1B (TNFRSF1B), nuclear factor kappa-B p65 subunit (NF-κBp65), and interferon-γ (IFN-γ) mRNA, while expression of NF-κB inhibitor-alpha (IκB-α) mRNA was significantly decreased [356]. Ginsenosides, the major constituents of ginseng with unique biological activities, were shown to promote proliferation of chicken primordial germ cells through protein kinase C (PKC)-involved activation of NF-κB [357].

Tanshinone IIA (TIIA), a major lipophilic component extracted from the root of *Salvia miltiorrhiza* Bunge used in Chinese medicine, was shown to have a protective effect against pulmonary arterial hypertension-related inflammatory responses [358]. Treatment with an extract of *Hypericum perforatum* L., also known as Saint John’s wort, at doses of 480–120 mg/kg for 5 days was shown to reduce infectious bronchitis virus (IBV)-induced injury and reduced the mRNA expression level of IBV in the chicken trachea in vivo. In particular, the expression of IL-6, TNF-α, and NF-κB was shown to be significantly decreased, but mitochondrial antiviral signaling gene, IFN-α, and IFN-β mRNA levels were shown to be significantly induced in vitro and in vivo [359].

### 7.4. Other Nutrients and Probiotics

Retinoic acid, an active vitamin A metabolite, was indicated to activate the PI3K/Akt and NF-κB signaling pathways, leading to proliferation of the cultured chicken primordial germ cells [360]. In LPS-challenged chickens, increased vitamin E supplementation (50 mg/kg vs. 10 mg/kg) was shown to decrease the expression of nuclear NF-κB p65 and increase the levels of IκBα in the liver [361]. It seems likely that the inhibitory effects of vitamin E on NF-κB expression could also be seen in physiological conditions, without any stress challenges. For example, in broilers fed increased vitamin E levels (14.11–14.91 mg/kg vs. 4.38–4.63 mg/kg) for 21 or 42 days, liver NF-κB p65 levels were significantly decreased, whereas liver IκB-α levels were significantly increased [362]. The NF-κB DNA-binding activity in the high-density housing group was shown to increase significantly compared with the free-range and low-density housing birds. Dietary taurine (0.1%) was shown to significantly alleviate NF-κB DNA-binding activity in chicken liver [363] and layer oviduct tissue [364], which was initially increased due to high-density housing systems. Interestingly, in the chicken renal tissue, the same dose of dietary taurine was able to decrease the NF-κB DNA-binding activity only in the low-density housing environment [365].

Necrotic enteritis (NE) infection was shown to significantly upregulate the mRNA levels of the immune-related molecules TLR-2, IL-1β, IL-4, IL-10, IFN-γ, and iNOS and the growth factors TGF-β3 and insulin-like growth factor 2 (IGF2) in the jejunum of broiler chickens. However, NF-κB expression was not affected. Interestingly, compared with nonsupplemented groups, probiotic *Enterococcus faecium* NCIMB 11,181 was shown to ameliorate necrotic enteritis-induced intestinal barrier injury in broiler chickens and increased gene expression levels of TLR2, MyD88, NF-κB, IL-4, iNOS, TGF-β3, PI3K, IGF-2, glucagon-like peptide-2 (GLP-2), and epidermal growth factor receptor (EGFR). There was also a significant interactive effect of NE infection and *E. faecium* treatment on the mRNA expression of various immune-inflammation factors, including NF-κB [366]. A dendritic cell (DC)-targeted mucosal vaccine using *Lactobacillus plantarum* as an antigen delivery system against G57 virus infection was developed. The vaccine was shown to confer efficient protection against G57 H9N2 infection, due to activation of DCs by the TLR-induced NF-κB pathway. This was associated with improved DC migration and improving the presentation of immunogen to T and B lymphocytes, causing changes in T-cell polarization toward Th1, Th2, and regulatory T cells (Treg cells) and inducing more efficient mucosal and adaptive immunity responses [367]. Chickens infected with *Salmonella enteritidis* (0.5 mL of *S. enteritidis* bacterial suspension, 10^9^ CFU/mL) through the oral cavity at 9 days of age) were administered with a probiotic *Pediococcus pentosaceus* microcapsule (1 g/kg). It was shown that the probiotic effects on proinflammatory indices were time-dependent; the probiotic significantly decreased the NF-κB expression in spleen and cecum samples at 1 dpi, whereas the difference disappeared at 3 dpi, and NF-κB expression was significantly increased in samples from chickens fed the probiotic at 7 dpi before disappearing again 14 dpi [368].

## 8. NF-κB and Inflammation in Poultry Production

Inflammation is known to be a highly orchestrated and tightly controlled protective mechanism dealing with infection and coordinating the repair and regeneration process. However, in the case of misregulation of inflammation or its chronic character, it can be responsible for severe tissue damage [369]. Therefore, elucidation of the molecular mechanisms underlying the regulation/control and resolution of inflammation is among the major topics of modern medical and veterinary sciences. Indeed, the relationship among inflammation, NF-κB signaling, and chronic diseases, including cancer [370], liver diseases [371], multiple sclerosis [372], immune-related disorders [69,373], and others [374], has received tremendous attention in recent years. Uncontrolled inflammation is shown to be associated with many widely occurring human diseases, including cardiovascular disease, neurodegenerative diseases, diabetes, obesity, asthma, arthritis, and periodontal diseases [375]. Therefore, modulating inflammation through the negative regulation of NF-κB signaling is an important approach in medical sciences and clinical practices [376].

In fact, the efficacy, duration, and outcomes of an inflammatory response in poultry were shown to be condition-dependent and determined by the triggering signal recognized by the innate immune receptors [377]. It seems likely that poultry metabolic diseases related to cardiovascular ailments, responsible for a major portion of the flock mortality, as well as musculoskeletal disorders responsible for slowing down chicken growth and causing lameness [378] and leg/bone disorders [379], are associated with increased and misregulated inflammatory responses. Intestinal health is known to depend on a range of host-related factors (immunity, mucosal barrier), as well as nutritional, microbial, and environmental challenges. Thus, intestinal dysbiosis, compromised mucosal barrier, and gut inflammation are among major issues in commercial poultry production systems after the ban on growth-promoting antimicrobials in animal feed [380]. Furthermore, non-nutritive dietary ingredients, biosecurity, immunology, vaccine technology, and genetics are suggested to play an important role in antibiotic-free poultry production [40].

It was hypothesized that subclinical gut disorders leading to a chronic low-level inflammatory response in the gut associated with oxidative stress and redox disbalance could result in the disruption of digestive function and poor immune competence [44]. Furthermore, the authors also suggested that chronic intestinal inflammation is responsible for the decreased performance and increased incidence of intestinal problems observed in poultry production. In fact, there are a range nutritional stress factors, including feed with a high content of nonstarch polysaccharides, crude protein excess, ingredient rancidity, or mycotoxin- and/or heavy-metal-contaminated feed, which could trigger gut inflammation in poultry species [30,44].

Inflammation and the susceptibility to inflammatory disorders are known to be regulated by nutrition, the gut microbiome, and genetics. In fact, altered nutrient status is shown to reprogram host inflammation via the gut microbiota [381]. Furthermore, the crosstalk between gut bacteria and host immunity is considered to be of great importance in intestinal inflammation [382], and the intestinal microbiota has emerged as a key player in metabolic inflammation and dysfunction [383]. The modulation of inflammatory responses associated with NF-κB regulation has been described for many nutrients, including selenium, taurine, carnitine, silymarin, and other nutrients [30]. Furthermore, commercially used feed additives, including probiotics, prebiotics, phytobiotics, organic acids, short- and medium-chain fatty acids, essential oils, and enzymes, can help maintain optimal host–microbiome interactions to support gut integrity and efficient growth and health [384,385].

NF-κB is known as a master regulator of the inflammatory response in the complex inflammatory network, playing a critical role in the host defense against pathogens, and it protects cells from apoptosis, as a result of triggering the upregulation of proinflammatory genes [386]. Furthermore, in acute inflammation, NF-κB, as a redox-sensitive transcription factor, was shown to participate in condition-dependent selective regulation of the expression of many target genes. They include proinflammatory cytokines (TNF-α, IL-1β, IL-6, and IL-12), various antioxidants (e.g., glutamate cysteine ligase, SOD1, SOD2, and catalase) [386], proinflammatory enzymes (secretory phospholipase 2, sPLA2; COX-2, and iNOS), chemokines (macrophage inflammatory protein 1α (IP-1α), monocyte chemoattractant protein-1 (MCP1), growth factors, cell-cycle regulatory molecules, anti-inflammatory molecules, and adhesion molecules [387]. Under stress conditions, the NF-κB network is responsible for balancing the effect of various intracellular stress signals to maintain homeostasis and cell/tissue integrity [30]. Therefore, a better understanding of the condition-dependent molecular events/mechanisms determining the point at which NF-κB responses switch from being protective to become damaging is of great importance [372,388] and awaits further investigation.

## 9. Conclusions

Today, NF-κB is known as a redox-sensitive, inducible nuclear transcription factor that regulates the expression of a number of genes associated with important biological processes, including innate and adaptive immune responses, cell growth, maturation, survival, and adaptive homeostasis establishment via interactions with other transcription factors and vitagenes [1]. Indeed, NF-κB signaling is an important element in cell adaptation to a diverse array of environmental stimuli, including oxidative stress. These stimuli can be recognized by various receptors, and the subsequent response involves specific adapter proteins. Under normal physiological conditions, the antioxidant defense network and the optimized redox balance in the cell/tissue are responsible for orchestrating important biological processes associated with cell protection, repair, and growth. This includes T-cell maturation, fighting against infections, DNA damage repair, and tissue healing and integrity restoration after injury. However, under various stress conditions, associated with redox disbalance, aberrant NF-κB activation can lead to detrimental changes, including the development of many age-related diseases. In fact, it was reported that activation of NF-κB is an important mechanism of host defense against infection and stress [77]. Indeed, the results from gene knockout experiments clearly showed that mice deficient in NF-κB-mediated transcriptional regulation were susceptible to a variety of infections and were characterized by compromised immunity: specifically depressed immunoglobulin expression, defective humoral immune responses, and decreased responses to LPS [389]. In fact, it was recently proposed that NF-κB is a vital regulator of inflammation, indicating that the dynamical attributes and the composition of the nuclear NF-κB complexes cumulatively instruct context-specific inflammatory gene patterns [48]. A variety of endogenous and exogenous stimuli in poultry have been characterized, including danger, damage, and survival signals from viral and bacterial components, proinflammatory cytokines, DNA damage, growth factors, and other stressors. As mentioned above, the stimuli can be recognized by a range of various receptors with subsequent activation of NF-κB, along with involvement of an array of specific adapter proteins [60,67].

Poultry production was shown to be associated with a range of stresses, which can be divided into four major categories, namely, environmental, technological, nutritional, and internal/biological stresses [28,29]. It was proven that RONS excess and a compromised antioxidant defense network are responsible for compromised health, as well as the decreased productive and reproductive performance of growing broilers, laying hens and breeder birds [30,390]. Therefore, the antioxidant defense network in poultry is of great importance, and understanding the molecular mechanisms underlying its regulation to optimize redox balance in various tissues and in the body in general under commercially relevant stress conditions is an important topic of current research in avian biology and poultry health [26,30].

Our analysis of the recent literature related to the regulatory roles of NF-κB in poultry can be summarized as follows:

- Similar to mammalian species, in poultry, NF-κB plays a central role in the regulation of many physiological and pathological processes.
- In thermally stressed birds, NF-κB expression is condition-dependent, including temperature, exposure duration, and bird’s age.
- The effects of dietary AFB1 on NF-κB expression in chicken liver are also condition-dependent. In general, AFB1 was shown to compromise AO defenses and increase proinflammatory cytokine production via NF-κB induction.
- Mn or Cu excess in the chicken diet was shown to increase the expression of NF-κB in testes, heart, and immune organs.
- The proinflammatory effects of heavy metals (As, Cd and Pb) in chickens were shown to be mediated via NF-κB pathway activation in various tissues.
- Increased concentrations of NH_3_ and H_2_S (main environmental stressors in poultry production) in air during chicken housing were shown to impose oxidative stress and inflammatory responses via NF-κB activation.
- The stimulating effect of LPS on NF-κB expression was shown in vitro in model systems and in vivo with poultry.
- In many bacterial and viral diseases, NF-κB is activated to increase proinflammatory cytokine production and impose an inflammatory response to create a hostile environment for pathogens.
- The main bacterial pathogens causing various diseases in poultry production, including *Escherichia coli*, various *Salmonella* species, *Mycoplasma gallisepticum, Eimeria tenella, Clostridium perfringens*, and *Chlamydia psittaci*, were shown to induce proinflammatory responses in birds associated with increased NF-κB expression and activity.
- In model systems based on an investigation of gene expression changes due to various infections, it was proven that the development of viral diseases, including infectious bursal disease, Newcastle disease, Marek’s disease, and reovirus challenges, was associated with the induction of NF-κB and inflammatory responses.
- Nutritional modulation of NF-κB expression and activity was shown to be achieved by using various antioxidants, including selenium, various polyphenols, taurine, retinoic acid, vitamin E, and some probiotics. In fact, under various stress conditions, these nutrients can ameliorate (partly or completely) the increased NF-κB expression and activity imposed by stressors.

An opportunity to use nutritional/pharmacological means to modulate NF-κB expression and activity should be exploited further to deal with inflammation-associated poultry disorders/diseases, including gut health problems associated with antibiotic-free poultry production. In fact, various nutrients, including taurine [391], carnitine [392,393,394], silymarin [352], vitamin E [21], selenium [13,20], carotenoids [22], and others [26], have been shown to affect antioxidant defenses and vitagenes, helping maintain the redox balance in various chicken tissues. There is a need for further research related to interactions of the antioxidant defense network with vitagenes and transcription factors, including NF-κB and Nrf2, which are responsible for the maintenance of redox homeostasis and stress adaptation under the commercial conditions of poultry production.

## Figures and Tables

**Table 1:** Possible mechanisms of Nrf2–NF-κB interactions.

Mechanisms of Nrf2–NF-κB Interactions	References
Inhibiting effects of Nrf2 on NF-κB	
Decreasing the intracellular ROS levels. This inhibits oxidative stress-mediated NF-κB activation	[103]
Preventing the IκB-proteasomal degradation and inhibiting nuclear translocation of NF-κB. In Nrf2-deficient cells an inhibitor of NF-kB activity (IκB) is over-phosphorylated with rapid proteasomal degradation and increased NF-κB activity. Upregulation of Nrf2 induces increase heme oxygenase-1 (HO-1) levels and induce phase II enzymes expression blocking the degradation of IκB	[104,105,106,107]
Reducing p50 and p65 DNA binding. Nrf2 silencing enhanced p50 and p65 DNA binding and tumour necrosis factor (TNF)-α-induced proinflammatory gene expression	[108]
Preventing the recruitment of RNA polymerase II to start transcription of NF-κB-regulated genes. Nrf2 binds to regulatory regions of proinflammatory genes in an antioxidant-response element (ARE)-independent manner and prevents the recruitment of RNA polymerase II to start transcription of NF-κB-regulated genes	[109]
Competition between Nrf2 and p65 for binding to the transcriptional co-activator CBP-p300 complex. Overexpression of p65 limits the availability of CBR for Nrf2 interaction. Knockdown of p65 promotes Nrf2 complex formation with CBR	[110,111]
Degrading IKKβ through ubiquitination by Keap1	[112]
Inhibiting effects of NF-κB on Nrf2	
Inactivating Nrf2 by inducing cyclooxygenase 2	[113,114]
Recruiting MAF BZIP Transcription Factor K (MafK)-associated histone deacetylase 3 (HDAC3) activity to the HO-1 enhancer and deacetylating CBP leading to a suppression of its co-activator activity	[115,116]
Interacting with CREB-binding protein, the competent Nrf2 coactivator, and inhibiting the transcription of genes regulated by Nrf2	[111,117,118]
Decreasing free CBP, a transcriptional co-activator of Nrf2, and promoting phosphorylation of p65. Overexpression of p65 limits the availability of CBR for Nrf2 interaction. Knockdown of p65 promotes Nrf2 complex formation with CBR	[111]
κB sites in proximal promoter of Nrf2 are believed to be subject to binding and transcription initiation by p65	[119]

## Abbreviations

AFB1	aflatoxin B1
AKT	protein kinase B
ALT	alanine aminotransferase
AO	antioxidant
AP1	transcription factor
APEC	avian pathogenic *Escherichia coli*
ARD	ankyrin repeat domain
ARE	antioxidant response element
ATF3	activating transcription factor 3
CAT	catalase
COX2	cyclooxygenase-2
CXCLi2	recombinant chicken chemotactic and angiogenic factor 2
Cyt C	cytochrome C
DC	dendritic cells
dpi	days post infection
HDAC3	histone deacetylase3
IBD	infectious bursal disease
IBDV	infectious bursal disease virus
IFIT5	Interferon-induced protein with tetratricopeptide repeats 5
iNOS	inducible nitric oxide synthases
IRF7	interferon regulatory factor 7
LOX5	lipoxygenase 5
NDV	Newcastle disease virus
PGC-1α	peroxisome proliferator-activated receptor coactivator
p53	tumor protein p53
PPAR	peroxisome proliferator-activated receptor
PTEN	phosphatase and tensin homolog
PUFAs	polyunsaturated fatty acids
ROS	reactive oxygen species
RNS	reactive nitrogen species
FHS	follicle-stimulating hormone
FOXO3	transcription factors
FOXP3	forkhead box protein P3
GCL	glutamyl-cysteine ligase
GCLC	catalytic subunit of GCL
GR	glutathione reductase
Grx	glutaredoxin
GSH	reduced glutathione
GPx	glutathione peroxidase
GST	glutathione *S*-transferase
HAT	histone acetyl transferase
HIF	hypoxia-inducible factor
HO-1	heme oxygenase 1
HS	heat stress
HSP	heat-shock protein
IκB	inhibitor of κB
IKK	IκB kinase
IL-1R	IL1 receptor
IFN-γ	interferon gamma
Keap1	Kelch-like erythroid cell-derived protein with CNC homology (ECH)-associated protein 1
LPS	lipopolysaccharide
MAPK	mitogen-activated protein kinase
MDA	malondialdehyde
MIP-1α	macrophage inflammatory protein
MG	*Mycoplasma gallisepticum*
MMP16	matrix metalloproteinase 16
Msr	methionine sulfoxide reductase
MT3	metallothionein 3
NEMO	the regulatory subunit IKKγ
NF-κB	nuclear factor-κB
NLR	NOD-like receptor
NRLC5	NOD-like receptor family CARD domain containing 5
NQO1	NAD(P)H:quinone dehydrogenase 1
Nrf2	nuclear factor-erythroid-2 (NF-E2) and related factor 2
PCNA	proliferating cell nuclear antigen
PGC-1α	peroxisome proliferator-activated receptor-gamma coactivator 1α
PPARs	peroxisome proliferator-activated receptors
Prx	peroxiredoxin
PTGEs	prostaglandin E synthase
PGE	prostaglandin E
PRR	pattern recognition receptor
RANKL	receptor activator of NF-κB ligand
RHD	Rel homology domain
RONS	reactive oxygen and nitrogen species
ROS	reactive oxygen species
SeMet	selenomethionine
SOD	superoxide dismutase
sPLA2	secretory phospholipase 2
STAT3	signal transducer and activator of transcription 3
TGF-β	transforming growth factor beta
Th1	T helper cell 1
TLR	Toll-like receptor
TNF-α	tumor necrosis factor
TNFR	TNF receptor
TRAF6	tumor necrosis factor receptor-associated factor 6
TRIF	TIR-domain-containing adapter-inducing interferon-β
Trx	thioredoxin
TrxR	thioredoxin reductase
UCP2	uncoupling protein 2
XOR	xanthine oxidoreductase
Wnt4	signaling molecule (Wingless-related MMTV integration site 4)
